# Monitoring kinetic changes and restriction of influenza A virus RNA species during infection using a Flu-Stranded CRISPR platform

**DOI:** 10.1128/mbio.01789-26

**Published:** 2026-09-14

**Authors:** Anh-Tu Tran, Wen-Fang Tang, Chung-Fan Hsieh, Ya-Fang Chiu, Chung-Guei Huang, Kuo-Ming Lee, Rei-Lin Kuo, Kuei-Ching Hsiung, Cheng-Hsun Chiu, Jim-Tong Horng

**Affiliations:** 1 Graduate Institute of Biomedical Sciences, Chang Gung University 210836, Taoyuan, Taiwan; 2 Department of Biochemistry and Molecular Biology, College of Medicine, Chang Gung University 71589, Taoyuan, Taiwan; 3 Research Center for Emerging Viral Infections, Chang Gung University 847150, Taoyuan, Taiwan; 4 Molecular Infectious Disease Research Center, Chang Gung Memorial Hospital at Linkou, Taoyuan, Taiwan; 5 Division of Infectious Diseases, Department of Medicine, Chang Gung Memorial Hospital, New Taipei, Taiwan; 6 Department of Microbiology and Immunology, Chang Gung University 56081, Taoyuan, Taiwan; 7 Department of Laboratory Medicine, Linkou Chang Gung Memorial Hospital 548158, Taoyuan, Taiwan; 8 International Master Degree Program for Molecular Medicine in Emerging Viral Infections, Chang Gung University 56081, Taoyuan, Taiwan; 9 Department of Medical Biotechnology and Laboratory Science, Chang Gung University 56081, Taoyuan, Taiwan; 10 Research Center for Chinese Herbal Medicine, College of Human Ecology, Chang Gung University of Science and Technology 684826, Taoyuan City, Taiwan; The Ohio State University, Columbus, Ohio, USA

**Keywords:** influenza A virus, CRISPR-Cas12a, strand-specific RNA detection, viral RNA kinetics, early infection, RIG-I, nuclear import, clinical diagnostics

## Abstract

Influenza A virus (IAV) generates three closely related RNA species: viral RNA (vRNA), complementary RNA (cRNA), and messenger RNA (mRNA), whose strand-specific quantification remains limited by sensitivity and quantitative dynamic range, particularly at low RNA abundance. Here, we developed Flu-Stranded CRISPR-Cas12a, a strand-specific detection platform integrating tagged reverse transcription, segment-specific PCR, and Cas12a collateral cleavage to support quantitative analysis of all three RNA species across a broad dynamic range. The assay enables reliable detection down to 10^2^ copies per reaction, extending the lower quantitative boundary relative to both SYBR Green and TaqMan reverse transcription quantitative PCR (RT-qPCR) under matched conditions. Validated in infected cell lines, murine lung tissues, and clinical nasopharyngeal specimens, the platform enabled subtype-discriminating, strand-resolved detection, including samples near or below the quantitative range of SYBR Green RT-qPCR. Using finely resolved infection time-course analyses in NP and NA segments, we identified a reproducible early vRNA decline within the early post-infection phase. This decline was partially attenuated in RIG-I knockout A549 cells, while subsequent vRNA accumulation was enhanced, consistent with a modulatory rather than essential role for RIG-I in early viral RNA dynamics. Subcellular fractionation localized this decline to cytoplasmic incoming genomes. In contrast, importazole-mediated inhibition of nuclear import abolished vRNA recovery without affecting the early decline, indicating that nuclear entry functionally separates early genome reduction from subsequent productive replication. These findings establish Flu-Stranded CRISPR-Cas12a as a strand-resolved framework for monitoring IAV RNA dynamics and reveal an early window of genome vulnerability during cytoplasmic transit that shapes infection outcome.

**IMPORTANCE:**

The early fate of incoming influenza virus genomes remains unclear, limiting our understanding of how infection is established or aborted in host cells. We developed Flu-Stranded CRISPR-Cas12a, a strand-specific platform for sensitive and quantitative analysis of influenza viral RNA (vRNA), complementary RNA (cRNA), and messenger RNA (mRNA) across experimental and clinical samples. Using high-resolution time-course analysis, we identified a reproducible early decline in vRNA during the post-entry phase. Our data suggest that this early genome loss arises from multiple processes, with RIG-I acting as a modulatory factor rather than a primary driver. Subcellular fractionation localized this effect to cytoplasmic incoming genomes, whereas importin-β-mediated nuclear entry was required for subsequent vRNA recovery. These findings support a model of an early cytoplasmic phase of genome attrition that is distinct from replication and provide a framework for understanding early influenza RNA kinetics and for guiding strand-resolved diagnostics and antiviral evaluation.

## INTRODUCTION

Influenza A virus (IAV), a segmented negative-sense RNA virus, continues to pose a major global health threat due to its rapid antigenic evolution, zoonotic potential, and capacity to evade host immune detection (1, 2). The viral genome consists of eight single-stranded RNA segments (vRNA), each packaged into a viral ribonucleoprotein complex (vRNP) composed of RNA-dependent RNA polymerase (RdRp) and multiple copies of nucleoprotein (NP), which tightly regulate viral genome replication and transcription (2). Upon entering host cells through endocytosis, these vRNPs are released into the cytosol from the acidified endosome, promoted by the viral M2 pH-gated proton channels, and after the fusion of the viral envelope with endosomal membranes mediated by viral hemagglutinin (3). Subsequently, vRNP complexes are translocated into the nucleus to initiate transcription and replication (3, 4). RdRp snatches the 5′ caps of nascent host messenger RNA (mRNA) to initiate the transcription, synthesizing polyadenylated mRNAs for protein production (3, 4). The newly synthesized viral proteins accumulate and activate the production of complementary RNAs (cRNAs), which serve as replication intermediates for generating progeny viral genomes (5, 6).

While viral mRNA levels increase immediately after IAV vRNA translocates into the nucleus and decrease after sufficient protein production and accumulation, cRNA levels rise and stabilize at a plateau because they are solely used as a template for producing vRNA (7–9). In contrast, vRNA levels continuously increase and accumulate throughout the infection (7–9). Thus, vRNA, cRNA, and mRNA constitute the molecular backbone, each fulfilling different roles at various stages of the IAV life cycle. Identifying each IAV RNA species and monitoring their expression kinetics enhances our understanding of viral replication dynamics, host restriction, and pathogenesis across different IAV strains. Meanwhile, understanding the kinetics and relative abundance of these viral RNA species, especially during the early stages of infection when the host antiviral response first activates, is essential for studying the IAV replication process, dissecting key restriction points, and clarifying the timing of viral synthesis and immune evasion (10). These insights are crucial for drug development and improving vaccine production; however, accurately measuring strand-specific IAV RNA species remains a technical challenge (11–13). The significant overlap among vRNA, cRNA, and mRNA, particularly in coding regions, limits the effectiveness of conventional detection methods and platforms (14–17). Techniques, such as northern blotting, RNase protection assays, and primer extension, offer limited strand discrimination and are not readily scalable (12, 14–17). Even more advanced methods, such as tagged reverse transcription quantitative PCR (RT-qPCR), suffer from background amplification and a limited dynamic range (14). These limitations have hindered our ability to capture transient RNA species, resolve early replication events, and accurately quantify low-copy targets during infection, or assess antiviral responses in a strand-specific manner.

The clustered regularly interspaced short palindromic repeats (CRISPR)-CRISPR associated protein (Cas) 12a (CRISPR-Cas12a) system has emerged as a powerful platform for nucleic acid detection, owing to its programmability, sensitivity, and versatility (18–20). Upon recognizing a specific target sequence via a guide RNA (crRNA), Cas12a is activated to catalyze the collateral cleavage of nearby single-stranded DNA molecules, a trans-cleavage activity that forms the basis for fluorescent or colorimetric readouts (18, 19). The cleavage efficiency primarily depends on the complementarity between the crRNA and the target, enabling single-nucleotide specificity, while a protospacer adjacent motif (PAM) is essential for effective recognition and activation (18, 19). When combined with pre-amplification methods, such as PCR, Cas12a-based systems significantly enhance the sensitivity and specificity of nucleic acid diagnostics (21, 22). These integrated CRISPR-Cas12a platforms have been successfully applied to pathogen detection, viral genotyping, and molecular surveillance in clinical settings, offering a flexible alternative to traditional qPCR-based approaches (22–30).

To address practical limitations in the sensitivity and quantitative range of strand-specific detection in influenza A virus research, we developed Flu-Stranded CRISPR-Cas12a, a workflow that integrates tagged strand-specific reverse transcription, segment-specific PCR amplification, and Cas12a collateral cleavage to quantify vRNA, cRNA, and mRNA within a single integrated assay. By combining strand-discriminating primer tags with guide-directed Cas12a detection, the platform was designed to distinguish closely related viral RNA species despite their extensive sequence overlap and to support quantitative analysis across a broad dynamic range, including low-abundance conditions where RT-qPCR workflows may show reduced resolution. The platform achieved reliable quantification down to 10^2^ copies per reaction, extending the lower quantitative boundary at least one order of magnitude beyond both SYBR Green and TaqMan RT-qPCR under matched experimental conditions. We applied this approach in infected cells, murine lung tissues, and clinical nasopharyngeal specimens, and used it to profile influenza A virus RNA kinetics at high temporal resolution. Through this approach, we sought to define how early viral RNA dynamics, host restriction, and nuclear entry interact during the initial stages of infection while establishing a sensitive, strand-resolved detection strategy.

## RESULTS

### Design of a Flu-Stranded CRISPR-Cas12a platform for specifically quantifying three RNA species of IAV

**Fig 1:**
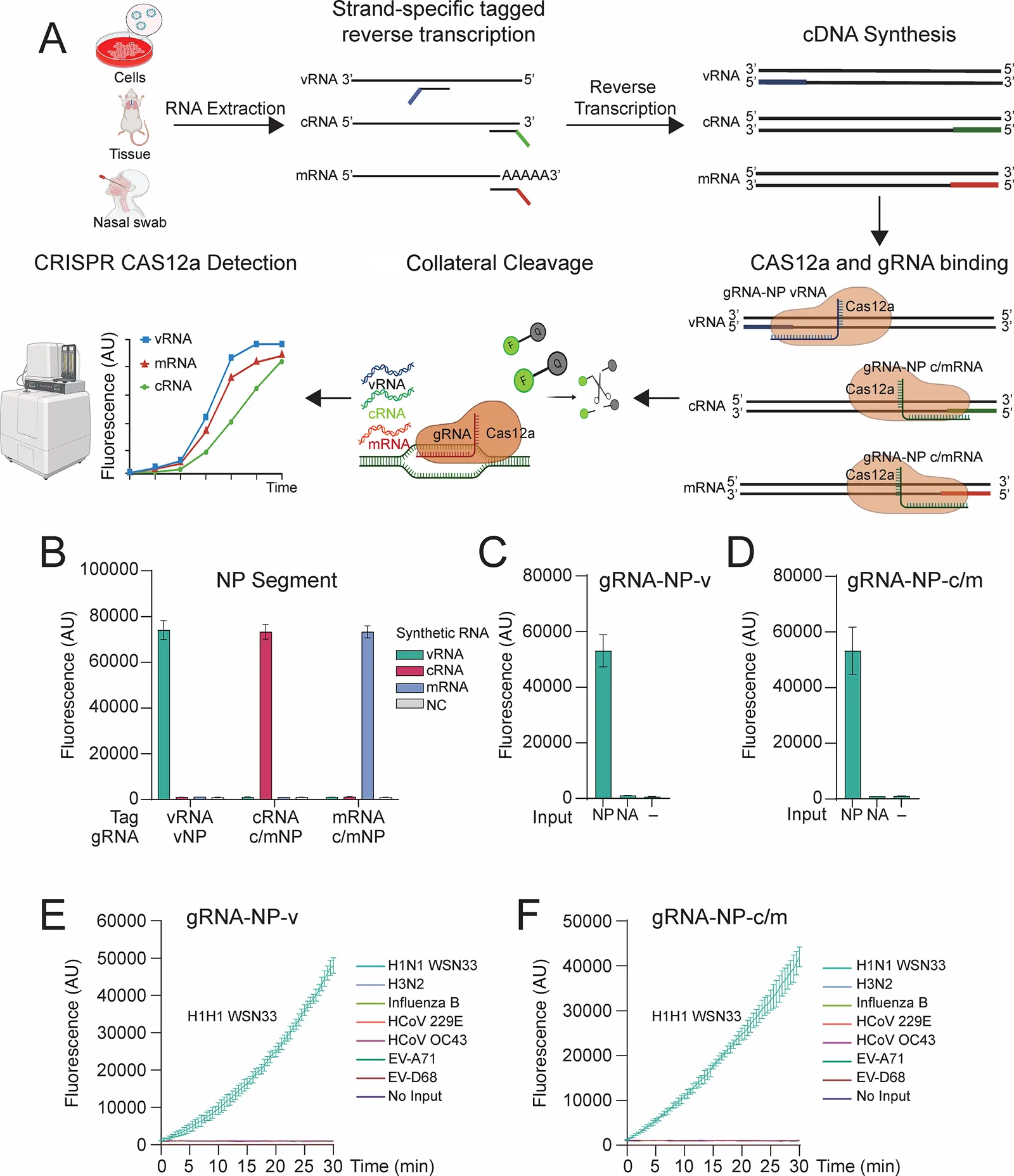
Schematic and experimental validation of a Flu-Stranded CRISPR-Cas12a detection platform for IAV RNA species. (**A**) Workflow diagram of the Flu-Stranded CRISPR-Cas12a assay. Total RNA from infected cells, tissue, or clinical swabs is reverse transcribed using strand-specific tagged primers for vRNA, cRNA, or mRNA. cDNAs are amplified via segment-specific PCR incorporating PAMs and detected using Cas12a collateral cleavage activity in the presence of strand-specific gRNAs, generating a fluorescent signal. (**B**) Strand specificity of gRNA-NP-v and gRNA-NP-c/m was validated using equimolar amounts of synthetic strand-specific NP vRNA, cRNA, and mRNA at a high-input concentration of 2 × 10^9^ copies per reaction, with copy numbers calculated from the theoretical molecular weight of each full-length transcript. To assess strand specificity in a biologically relevant context, reactions were additionally supplemented with 1 µg of total cellular RNA from mock-infected cells while holding IVT target input constant, with mock-RNA-only reactions serving as negative controls. Fluorescence was measured at 30 min and is expressed as arbitrary units (AU). (**C and D**) Validation of strand-resolved detection of Flu-Stranded CRISPR-Cas12a assay using synthetic vRNA, cRNA, or mRNA of the NP or NA with gRNA-NP-v (**C**) and gRNA-NP-c/m (**D**). Data represent mean ± SD from three independent experiments. (**E and F**) Pathogen specificity analysis of gRNA-NP-v (**E**) and gRNA-NP-c/m (**F**), tested against a panel of respiratory viruses, including H1N1, H3N2, influenza B, human coronaviruses (229E, OC43), and enteroviruses (EV-A71, EV-D68). Total RNA input was normalized to 500 ng per reaction. Data represent mean ± SD from three independent experiments.

IAV replication involves three structurally related yet functionally distinct RNA species, viral vRNA, cRNA, and mRNA, each playing a central role in the viral life cycle. mRNA supplies enough proteins for viral genome synthesis and progeny assembly; cRNA must be stable enough to serve as a template for producing progeny genomes; newly synthesized vRNA dictates the number of progeny produced (7, 8, 15). Therefore, determining the relative amounts of each IAV RNA species is crucial for understanding the propagation kinetics and characteristics of emerging IAV and even for designing therapeutic drugs. However, the extensive sequence overlap among these RNA species, especially between cRNA and mRNA, has posed a longstanding challenge in achieving strand-specific detection using conventional molecular tools (14–17). To overcome this limitation, we developed a platform by rationally combining CRISPR-Cas12a technology with PCR amplification using tagged primers to detect and quantify individual IAV RNA strands with high specificity and sensitivity across two representative viral gene segments: NP (segment 5) and NA (segment 6) of the pandemic human IAV H1N1 strains. Three key components are integrated into our assay (Fig. 1A): (i) reverse transcription using strand-specific tagged primers to generate uniquely labeled complementary DNAs (cDNAs) corresponding to vRNA, cRNA, or mRNA; (ii) segment-specific PCR amplification incorporating Cas12a-compatible PAMs; and (iii) Cas12a-mediated collateral cleavage to generate a fluorescence readout. For each segment, two guide RNAs (gRNAs) were designed: one specific for the vRNA (negative-sense strand), and another targeting a conserved coding region shared by both cRNA and mRNA (positive-sense strands). Given the high sequence identity between cRNA and mRNA, particularly within the open reading frame, strand identity is not determined by gRNA sequence alone but is resolved through primer tagging during the reverse transcription step, enabling precise attribution of signal to a specific RNA species.

To evaluate the strand specificity of our CRISPR-Cas12a assay, synthetic RNA templates corresponding to vRNA, cRNA, and mRNA of the NP and NA gene segments were transcribed *in vitro* and tested under high-input conditions (2 × 10^9^ copies per reaction for each RNA species). Cross-reactivity was assessed with fluorescence quantified at 30 min after Cas12a activation. Under these conditions, fluorescence signals were detected only when the tagged cDNA strand matched the target specified by the corresponding guide RNA. The results showed that gRNA designed to recognize NP vRNA (gRNA-NP-v) produced a strong fluorescent signal only in the presence of vRNA-tagged input, while gRNA recognizing NP cRNA or mRNA (gRNA-NP-c/m) selectively detected templates derived from cRNA or mRNA (Fig. 1B; Fig. S1A). No detectable signal was observed in mismatched guide-template combinations, non-cognate strand inputs, or negative controls, supporting the specificity of the strand-specific primer tagging strategy in combination with guide-directed Cas12a detection (Fig. 1B; Fig. S1A).

To further test whether strand specificity is maintained in a biologically relevant RNA background, increasing amounts of total cellular RNA from mock-infected cells, ranging from 0 to 1 µg, were added to a constant amount of IVT target RNA representing the cognate viral strand (Fig. S2). For both NP and NA targets, cognate strand detection remained robust and consistent across all background RNA concentrations, whereas non-cognate strand signals remained indistinguishable from mock-RNA-only negative controls. These findings indicate that endogenous host RNA did not measurably compromise strand specificity or produce detectable off-target Cas12a activation under the conditions tested, supporting the use of the platform with total RNA derived from infected cells, tissues, and clinical specimens.

Notably, gRNAs targeting NP exhibited no cross-reactivity with NA-derived templates, and vice versa (Fig. 1C and D; Fig. S1B and C), highlighting the segment specificity of our newly developed assay. These results confirm that the reverse transcription tagging-PCR combined with the Cas12a-mediated gRNA system (Flu-Stranded CRISPR-Cas12a) enables precise and orthogonal discrimination of both strand types and genomic segments.

To further evaluate the pathogen specificity of the system, we tested the assay against total RNA extracted from a panel of respiratory viruses, including influenza A/Hong Kong/1/1968 (H3N2) subtype, influenza B B/TW/99/07 virus, human coronavirus 229E and OC43, as well as other nonrespiratory viruses, such as enterovirus A71 (EV-A71) and enterovirus D68 (EV-D68). The results showed that, except for the H1N1 WSN IAV strain, neither other respiratory viruses nor nonrespiratory viruses generated a detectable signal, confirming that the assay provides high subtype fidelity and minimal off-target activation (Fig. 1E and F; Fig. S1D and E).

### Highly sensitive detection of IAV RNA species across a broad quantitative range using Flu-Stranded CRISPR-Cas12a

**Fig 2:**
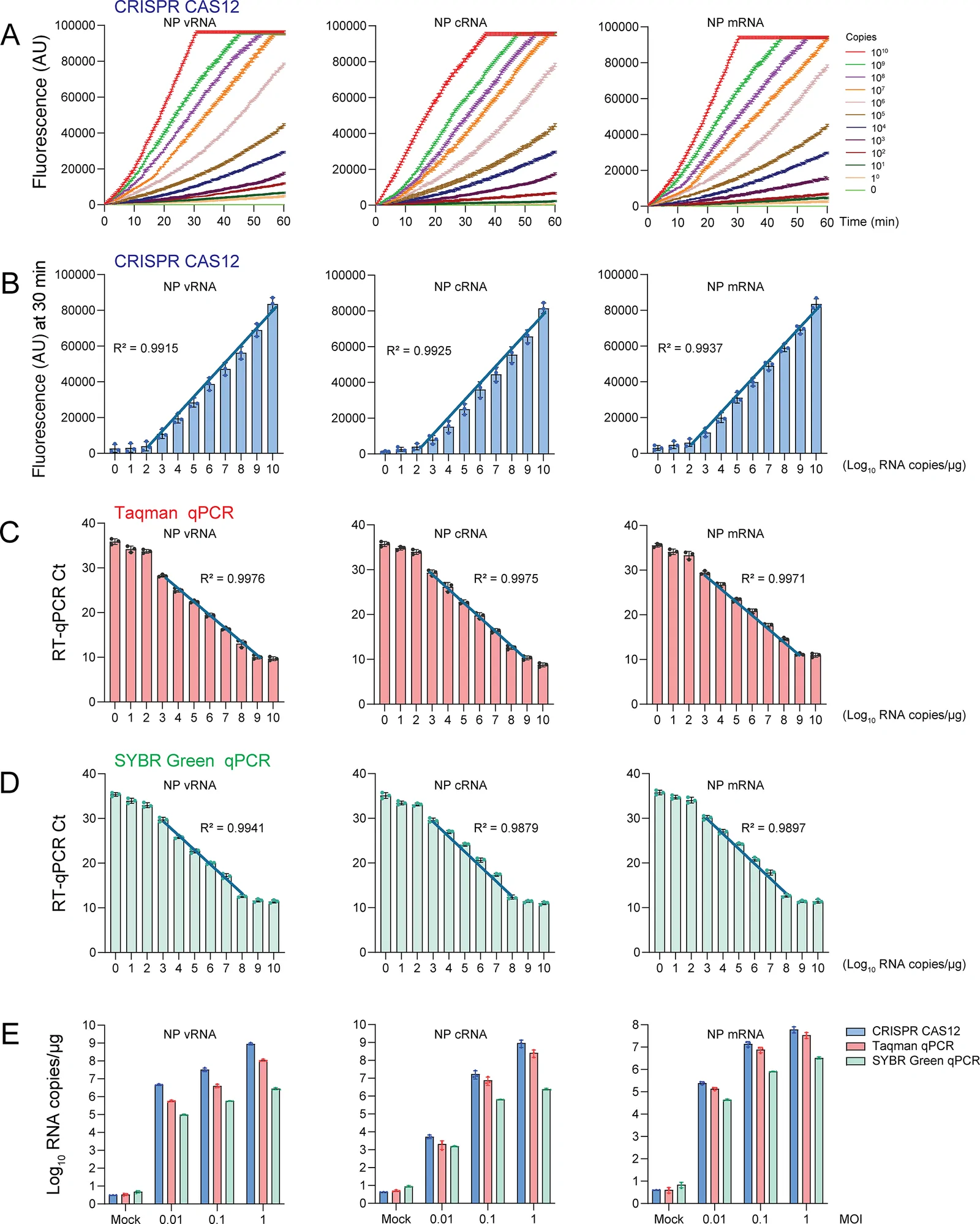
Flu-Stranded CRISPR-Cas12a enables high-sensitivity, strand-specific detection of NP vRNA, cRNA, and mRNA across a broad quantitative range. A serial 10-fold dilution series (10^10^ to 10^0^ copies per reaction) of synthetic strand-specific NP vRNA, cRNA, or mRNA was processed through the complete detection workflow and quantified using the Flu-Stranded CRISPR-Cas12a assay (**A and B**), TaqMan RT-qPCR (**C**), or SYBR Green RT-qPCR (**D**). (**A**) Real-time fluorescence signals generated by the Flu-Stranded CRISPR-Cas12a assay for NP vRNA, cRNA, and mRNA across the indicated RNA input range (10^10^ to 10^0^ copies per reaction). (**B**) Standard curves generated from Cas12a fluorescence measured at 30 min, plotted against log_10_ transformed input RNA copy number to evaluate assay linearity and dynamic range. (**C and D**) Standard curves for TaqMan RT-qPCR and SYBR Green RT-qPCR, respectively, generated by plotting Ct values against log_10_ transformed input RNA copy number for the same strand-specific RNA standards. The fitted line in each panel represents linear regression calculated using data points within the experimentally validated quantitative range. The validated quantitative ranges were 10^2^ to 10^10^ copies for Flu-Stranded CRISPR-Cas12a, 10^3^ to 10^9^ copies for TaqMan RT-qPCR, and 10^3^ to 10^8^ copies for SYBR Green RT-qPCR. Data points showing signal compression or reduced quantitative resolution at the lower or upper boundaries were excluded from the regression analysis. Data in panels **A–D** represent mean ± SD from three independent experiments. (**E**) Comparison of NP vRNA, cRNA, and mRNA levels in Madin-Darby canine kidney (MDCK) cells infected with A/WSN/1933 (H1N1) at multiplicities of infection (MOIs) of 0.01, 0.1, and 1. Total RNA was collected at 8 h post-infection (hpi), and the three NP RNA species were quantified in parallel using Flu-Stranded CRISPR-Cas12a (blue), TaqMan RT-qPCR (red), and SYBR Green RT-qPCR (green). RNA abundance is presented as mean ± SD from three independent experiments and expressed as log_10_ RNA copies per µg total RNA.

To determine the utility of our detection platform for quantifying different IAV RNA species and its sensitivity, the *in vitro*-transcribed vRNA, cRNA, and mRNA of the NP and NA segments were serially diluted in 10-fold increments (10^10^ to 10^0^ copies), followed by reverse transcription using strand-specific tagged primers. Following PCR amplification, the amplicons were subjected to Cas12a-mediated trans-cleavage, generating fluorescence readouts measured at 1 min intervals over 60 min. Flu-Stranded CRISPR-Cas12a generated clear copy-dependent fluorescence kinetics for NP vRNA, cRNA, and mRNA (Fig. 2A). Although fluorescence traces became less separated at higher input concentrations, likely due to partial saturation of the Cas12a collateral cleavage reaction, quantification at a fixed pre-saturation time point of 30 min preserved a strong linear relationship between fluorescence intensity and input RNA copy number across the quantifiable range. At this fixed time point, endpoint fluorescence intensity exhibited a strong linear correlation with input copy number (R^2^ ≥ 0.99 across three independent biological replicates), with quantitative response maintained down to approximately 10^2^ copies per reaction under our assay conditions (Fig. 2B). These data establish a quantitative range spanning approximately eight orders of magnitude (10^2^ to 10^10^ copies) for each NP RNA species. Comparable fluorescence kinetics and endpoint linearity were observed for the NA segment targets (Fig. S3A and B), with R^2^ values consistently above 0.98 across independent experiments, supporting the reproducibility of the standard curves used for downstream quantification.

The same dilution series was quantified in parallel by TaqMan RT-qPCR and SYBR Green RT-qPCR, both of which showed the expected linear Ct behavior over narrower validated ranges under the same template conditions (Fig. 2C and D). SYBR Green RT-qPCR remained quantitative across approximately five orders of magnitude (10^3^–10^8^ copies) for the NP RNA species (Fig. 2D), with similar results for NA (Fig. S3C). TaqMan RT-qPCR extended this range to approximately six orders of magnitude (10^3^–10^9^ copies) under the conditions tested (Fig. 2C). At the low-copy end of the dilution series, Flu-Stranded CRISPR-Cas12a maintained more consistent quantitative separation between adjacent inputs, whereas both RT-qPCR workflows showed reduced separation near their lower quantitative limit (Fig. 2B through D). These analytical benchmarks provide the context for interpreting assay performance in the subsequent infection time-course experiments.

To evaluate the platform in a biological context, Madin-Darby Canine Kidney (MDCK) cells were infected with A/WSN/1933 (H1N1) at multiplicities of infection (MOI) of 0.01, 0.1, and 1. Total RNA was isolated at 8 h post-infection (hpi), followed by strand-specific reverse transcription and quantification of NP (Fig. 2E) and NA (Fig. S3D) RNA species using both Flu-Stranded CRISPR-Cas12a and RT-qPCR. Viral RNA levels increased in an MOI-dependent manner across all measured species. For NP, vRNA levels increased from approximately 10^6^ copies/μg RNA at MOI 0.01 to nearly 10^9^ copies/μg RNA at MOI 1 as measured by Flu-Stranded CRISPR-Cas12a (Fig. 2E). A similar trend was observed for NA, with vRNA levels increasing from approximately 10^5^ to 10^8^ copies/μg RNA across the same MOI range (Fig. S3D). cRNA and mRNA followed comparable MOI-dependent trends (Fig. 2E; Fig. S3D). Across targets and MOIs, Flu-Stranded CRISPR-Cas12a consistently yielded higher apparent copy-number estimates than either TaqMan or SYBR Green RT-qPCR for NP RNA (Fig. 2E). Despite these differences in absolute values, all platforms showed concordant MOI-dependent trends. Similar behavior was observed for NA RNA (Fig. S3D). Together, these results demonstrate that Flu-Stranded CRISPR-Cas12a enables strand-specific quantification of influenza RNA across a range of infection conditions while preserving consistent relative trends with RT-qPCR.

### Flu-Stranded CRISPR-Cas12a enables sensitive *in vivo* detection of IAV RNA and resolves strand-specific kinetics in mice lungs

**Fig 3:**
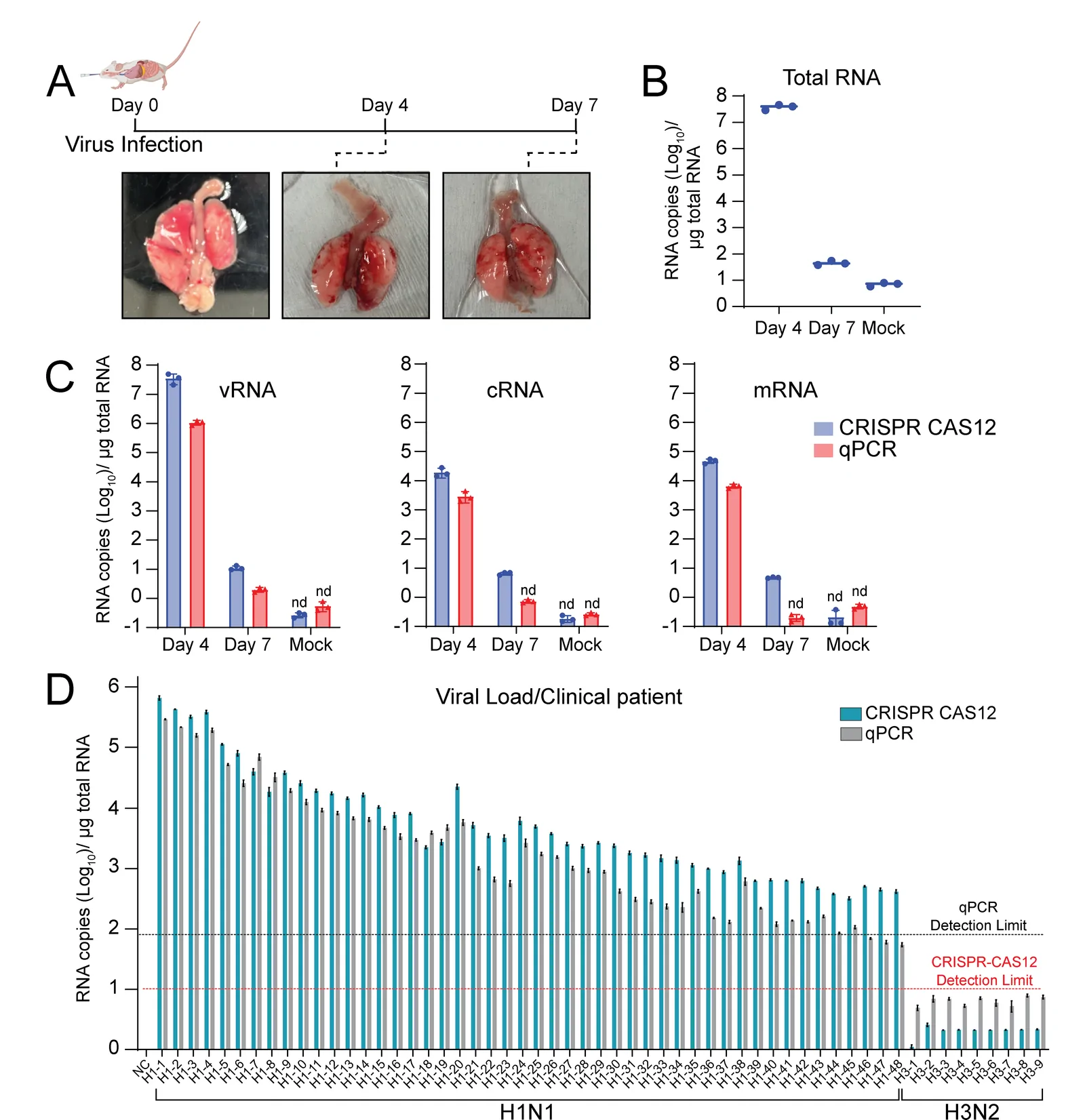
Flu-Stranded CRISPR-Cas12a enables strand-specific detection of IAV RNA species in murine lung tissue and clinical nasopharyngeal swab samples. Representative images of gross lung pathology in BALB/c mice (*n* = 3 per group) infected intranasally with influenza A/WSN/1933 (H1N1) (9 × 10^3^ PFU) and collected at 4 and 7 dpi (**A**). Total RNA was isolated from mock- and virus-infected lungs in mice at 4 and 7 dpi and quantified using conventional RT-qPCR (**B**). The NP segment vRNA, cRNA, and mRNA in the lung homogenates were further reverse transcribed with tagged strand-specific primers and determined by Flu-Stranded CRISPR-Cas12a detection (purple) or RT-qPCR analysis (pink) (**C**). Data represent mean ± SD from three mice per group. (**D**) Viral RNA was isolated from a group of 58 nasopharyngeal swabs collected from clinical patients diagnosed with influenza infections, with virus strains confirmed by RT-qPCR, including 48 H1N1-positive samples, 9 H3N2-positive controls (non-target subtype), and one from a healthy individual as a negative control (NC). Following strand-specific reverse transcription, the NP vRNA levels in these clinical samples were determined using Flu-Stranded CRISPR-Cas12a (blue bars) as compared to RT-qPCR (gray bars). vRNA levels are expressed as copies per μg of total RNA. Bars are ordered by decreasing viral load; dotted lines indicate the detection thresholds for each method.

Following the *in vitro* validation of our Flu-Stranded CRISPR-Cas12a assay, we next applied this platform to an *in vivo* murine model to monitor viral RNA kinetics in the respiratory tract following IAV infection. Six-week-old BALB/c mice were intranasally infected with 9 × 10^3^ plaque-forming units (PFU) of A/WSN/1933 (H1N1), and lung tissues were collected at 4 and 7 days post-infection (dpi), representing peak and resolution phases of infection, respectively (Fig. 3A). Gross pathological examination revealed pronounced lung inflammation and consolidation at 4 dpi, consistent with high viral replication. By 7 dpi, these lesions were largely resolved, indicating active immune-mediated clearance (Fig. 3A). Total RNA was extracted from infected and mock-treated lungs, and viral burden was quantified using both RT-qPCR and Flu-Stranded CRISPR-Cas12a platform. qPCR measurements of total influenza RNA demonstrated a marked decrease in viral load from 4 to 7 dpi, with mock controls showing no detectable signal (Fig. 3B). Quantification by both Flu-Stranded CRISPR-Cas12a and RT-qPCR revealed high levels of vRNA, cRNA, and mRNA at 4 dpi (Fig. 3C), reflecting active genome replication and transcription. In mock-infected controls, all three RNA species were undetectable, confirming the assay’s specificity. By 7 dpi, all three RNA species declined substantially. Notably, Flu-Stranded CRISPR-Cas12a retained detectable signals for low-abundance RNAs, particularly cRNA and mRNA, whereas qPCR frequently fell below the detection threshold (Fig. 3C). This differential sensitivity underscores the enhanced detection capability of Flu-Stranded CRISPR-Cas12a, particularly in post-acute or low-titer settings. The strong agreement between strand-specific Cas12 signal reduction and histopathological recovery further supports the biological relevance of the assay. While qPCR showed reliable detection over a dynamic range of 10^3^ to 10^8^ copies, Flu-Stranded CRISPR-Cas12a extended detection to lower RNA input levels to 10^2^ copies (Fig. 2; Fig. S3). These findings demonstrate that the Flu-Stranded CRISPR-Cas12a platform is comparable to, and in certain contexts more sensitive than, RT-qPCR, particularly for resolving residual viral RNA during the resolution phase of infection. Collectively, these data establish Flu-Stranded CRISPR-Cas12a as a highly sensitive, strand-specific platform for *in vivo* monitoring of IAV RNA species, with broad implications for preclinical pathogenesis studies, evaluation of antiviral efficacy, and clinical diagnostics.

### Flu-Stranded CRISPR-Cas12a enables sensitive and specific IAV detection in clinical samples

To evaluate the clinical utility of Flu-Stranded CRISPR-Cas12a, we analyzed a cohort of 58 nasopharyngeal swab specimens, including 48 H1N1-positive samples, 9 H3N2-positive samples as non-target subtype controls, and one negative control. Total RNA was extracted from all samples and analyzed using the Flu-Stranded CRISPR-Cas12a platform in a blinded manner, with comparison to routine RT-qPCR-based clinical diagnostic results. Following unblinding, subtype classifications obtained by Flu-Stranded CRISPR-Cas12a were fully concordant with the diagnostic assignments provided by the reference laboratory. Across the H1N1-positive samples, the assay produced detectable signals spanning a broad range of viral loads (Fig. 3D). Although absolute values differed between platforms, CRISPR-derived measurements showed concordant trends with RT-qPCR results, preserving relative sample ranking across the cohort. Notably, Flu-Stranded CRISPR-Cas12a maintained detectable signals in a subset of low-signal samples near the RT-qPCR detection threshold, consistent with its analytical sensitivity under low-input conditions established in benchmarking experiments (Fig. 2). To assess subtype specificity, the nine H3N2-positive samples and the negative control were analyzed. Under these conditions, Flu-Stranded CRISPR-Cas12a did not generate false-positive H1N1 signals, demonstrating subtype-level specificity consistent with RT-qPCR classification (Fig. 3D). Together, these results show that Flu-Stranded CRISPR-Cas12a enables accurate subtype classification and maintains concordance with RT-qPCR-based diagnostic frameworks across a wide range of viral loads in clinical specimens.

### High-resolution kinetics of IAV RNA species across all infection stages, followed by Flu-Stranded CRISPR-Cas12a

**Fig 4:**
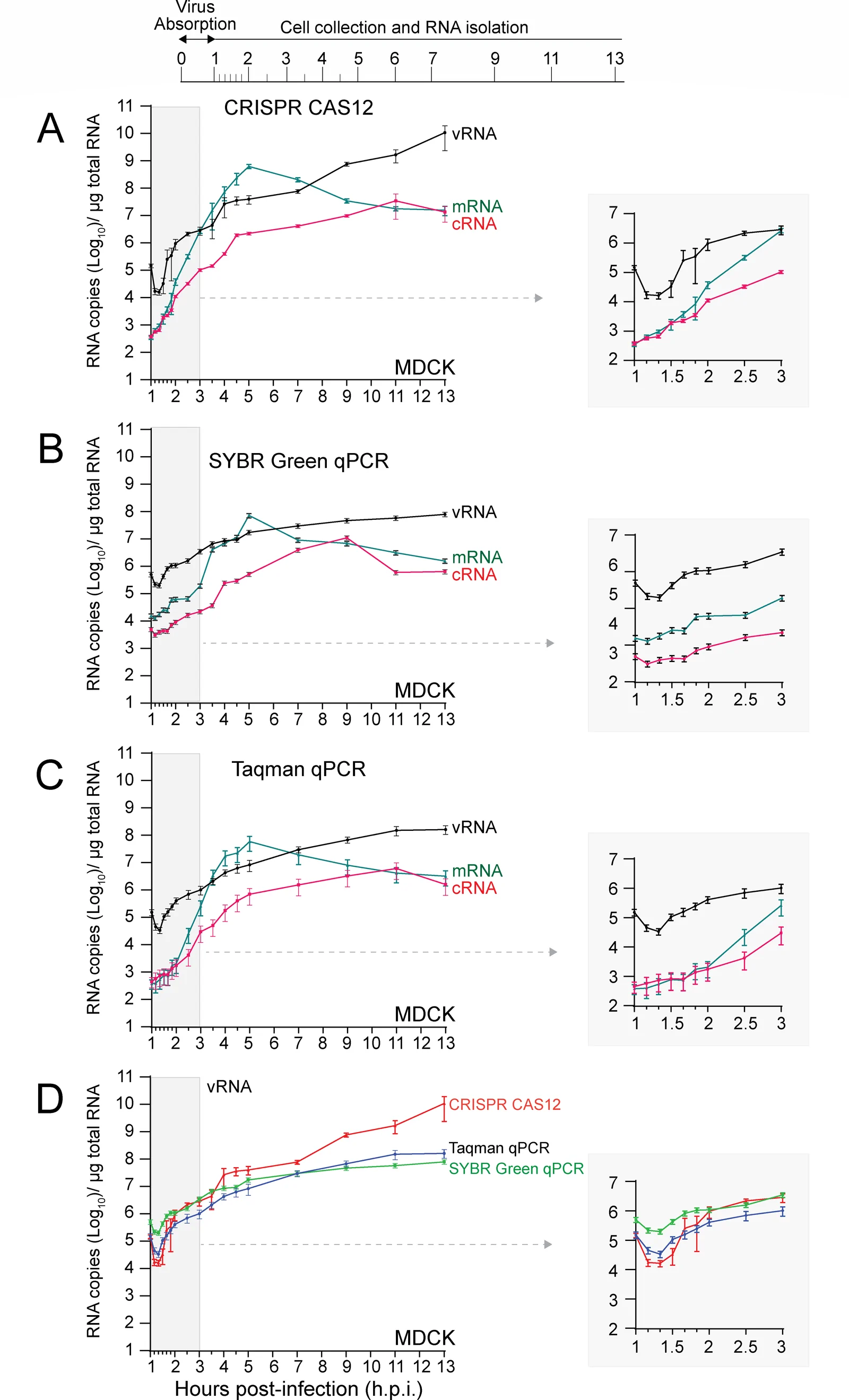
Comparative temporal profiling of influenza A virus NP RNA species using Flu-Stranded CRISPR-Cas12a and RT-qPCR platforms. Strand-specific kinetics of NP vRNA, cRNA, and mRNA were measured in MDCK cells infected with influenza A/WSN/1933 (H1N1) at an MOI of 10. Total cellular RNA was harvested at the indicated time points from 1 to 13 hpi. RNA species were quantified in parallel using the Flu-Stranded CRISPR-Cas12a assay (**A**), SYBR Green-based RT-qPCR (**B**), and TaqMan RT-qPCR (**C**). For each panel, the left subpanel depicts the full infection time course (1 to 13 hpi), and the right subpanel shows an expanded view of the early phase (1 to 3 hpi) to resolve early-phase kinetics. RNA abundance is presented as log_10_ copies per µg total RNA. (**D**) Direct overlay comparison of NP vRNA kinetics measured by Flu-Stranded CRISPR-Cas12a, SYBR Green RT-qPCR, and TaqMan RT-qPCR in MDCK cells. Data represent mean ± SD from three independent biological replicates.

To resolve the temporal dynamics of IAV replication with strand-level precision, we applied the Flu-Stranded CRISPR-Cas12a detection platform to quantify vRNA, cRNA, and mRNA across the course of infection. MDCK and A549 cells were infected with A/WSN/1933 (H1N1) at an MOI of 10, and total RNA was collected at multiple time points from 1 to 13 hpi. In parallel, the same RNA samples were quantified by Flu-Stranded CRISPR-Cas12a (Fig. 4A), SYBR Green RT-qPCR (Fig. 4B), and TaqMan RT-qPCR (Fig. 4C), enabling direct cross-validation of RNA kinetic profiles and assay performance across platforms.

The readouts from Flu-Stranded CRISPR-Cas12a profiling revealed that while NP and NA mRNA progressively increase and accumulate after IAV infection, their abundance peaks at approximately 5 hpi and then either gradually decline or stabilize in both MDCK and A549 cells (Fig. 4A; Fig. S4 and S5). These patterns reflect a shift from transcriptional dominance to genome replication. cRNA levels follow a similar trend but plateau slightly earlier, in line with its role as an intermediate in the replication process. Comparable temporal patterns of mRNA and cRNA expression for both NP and NA segments were observed by RT-qPCR, with differences in absolute values consistent with assay-dependent calibration (Fig. 4B and C; Fig. S4 and S5). These patterns are consistent with a transition from early transcriptional activity to genome replication at later stages of infection. In contrast, NP-derived vRNA levels exhibited a transient decline during the early phase of infection, between approximately 60 and 90 min post-infection (mpi), followed by a rebound to baseline by 2 hpi in MDCK cells (Fig. 4A). A similar early vRNA dip was observed for the NA segment (Fig. S4) and was consistently reproduced in A549 cells for the NP and NA segments (Fig. S5A and B). Following recovery, vRNA levels increased rapidly, reaching approximately 10^8^ copies per μg by 4–5 hpi in both MDCK and A549 cells. In contrast to the plateaued or modestly declining cRNA and mRNA at later time points, vRNA continued to accumulate throughout the late phase of infection (4 to 13 hpi) in both cell lines (Fig. 4A through C; Fig. S4 and S5).

To directly compare assay performance, RNA kinetics measured by Flu-Stranded CRISPR-Cas12a, SYBR Green RT-qPCR, and TaqMan RT-qPCR were plotted in parallel (Fig. 4D). Across the infection time course, all three methods showed concordant temporal patterns, including the transient early decline in vRNA, subsequent recovery, and continued accumulation at later time points. Absolute copy-number estimates differed between platforms. At mid and late stages of infection (3–13 hpi), Flu-Stranded CRISPR-Cas12a consistently yielded higher apparent values than both SYBR Green and TaqMan RT-qPCR. During the early post-adsorption window (1–3 hpi), differences between platforms were more pronounced at low RNA abundance. Despite these differences, all methods captured the same overall vRNA dynamics. Notably, the magnitude of this early decline was more clearly resolved by Flu-Stranded CRISPR-Cas12a compared with SYBR Green and TaqMan RT-qPCR (Fig. 4D), indicating improved resolution of fold-change magnitude under low-abundance conditions. TaqMan RT-qPCR showed closer agreement with Cas12a measurements than SYBR Green RT-qPCR across the time course, reproducing the same temporal pattern of early decline and recovery. Together, these results demonstrate consistent viral RNA kinetics across platforms, with reproducible patterns observed for vRNA, cRNA, and mRNA across NP and NA segments and in both MDCK and A549 cells (Fig. 4; Fig. S4 and S5).

### Live-cell imaging confirms cytoplasmic vRNA suppression during early IAV infection

**Fig 5:**
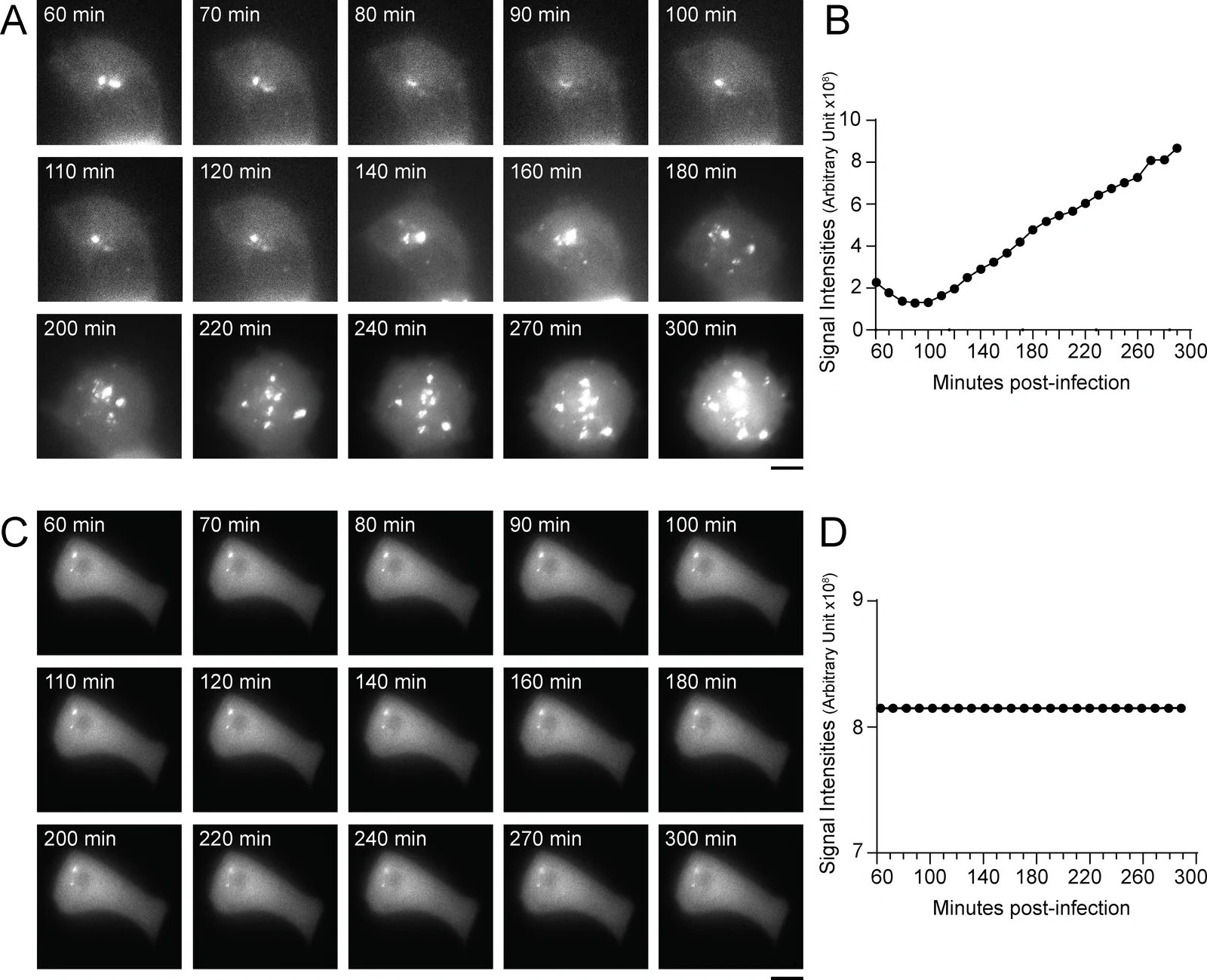
Live-cell imaging of IAV vRNA dynamics reveals early suppression phase following infection. (**A**) Time-lapse imaging of vRNA puncta in HEK293T cells co-transfected with MCP-GFPdN and pHH-PB2-vMSL, followed by infection with A/WSN/1933 (H1N1) at an MOI of 10. After 1 h of adsorption, live cell images were captured every 10 min for 4 h using a Nikon TiE Eclipse inverted microscope. Images show maximum intensity projections across z-stacks for a representative cell at the indicated time points. Scale bar, 5 μm. (**B**) Quantitative analysis of vRNA puncta in a representative cell across the 4 h imaging period. The number and fluorescence intensity of cytoplasmic vRNA puncta were extracted using the CAPS program and then counted, summed up, and plotted over time. Fluorescence values are reported in arbitrary units (AU × 10^**8**^). (**C**) Time-lapse imaging of a representative mock-infected HEK293T cell co-transfected with pMCP-GFPdN and pHH-PB2-vMSL under identical conditions, serving as a negative control for infection-dependent puncta formation. Scale bar, 5 μm. (**D**) Quantitative analysis of fluorescence signal in the representative mock-infected cell shown in panel **C**, showing stable diffuse signal without puncta accumulation throughout the imaging period. Fluorescence values are reported in arbitrary units (AU × 10^8^).

To validate the vRNA kinetics observed using Flu-Stranded CRISPR-Cas12a at the single-cell level, we employed a live-cell imaging strategy based on our MS2-MCP system (31). A reporter construct, pHH-PB2-vMSL, was generated by inserting 24 tandem MS2 stem-loop (MSL) sequences into the PB2 coding region, enabling a reporter vRNA (PB2-vMSL) transcribed from the pol I promoter to mimic viral vRNA (31). HEK293T cells were co-transfected with this construct and a plasmid encoding the MS2 coat protein fused to GFP lacking a nuclear localization signal (MCP-GFPdN), allowing for real-time visualization of cytoplasmic vRNA as fluorescent puncta. This system was used to monitor whether the early vRNA decline observed by Flu-Stranded CRISPR-Cas12a could also be detected at the single-cell level. Following infection with influenza A/WSN/1933 at an MOI of 10, live-cell fluorescence microscopy was performed to monitor vRNA dynamics. Among the 10 cells tracked over time, puncta number and fluorescence intensity of vRNA puncta were quantified using the Computer-Assisted Puncta Summation (CAPS) program (measured in arbitrary units, AU) (Table S1). During the first 60–120 mpi, puncta were few and showed reduced fluorescence intensity, indicating decreased cytoplasmic vRNA accumulation (Fig. 5A and B; Table S1). From 120 mpi onward, both puncta count and signal intensity increased markedly, suggesting the start of active replication (Fig. 5A and B; Table S1). In contrast, mock-infected cells expressing PB2-vMSL and MCP-GFPdN showed sparse and stable cytoplasmic GFP puncta over time, with no systematic changes in puncta number or fluorescence intensity (Fig. 5C and D; Table S2). These single-cell imaging results closely mirrored the vRNA kinetic profiles captured at the population level using Flu-Stranded CRISPR-Cas12a, independently supporting a transient early suppression phase, followed by strong amplification.

### Early influenza vRNA decline occurs before robust interferon activation and is only partially dependent on RIG-I signaling

**Fig 6:**
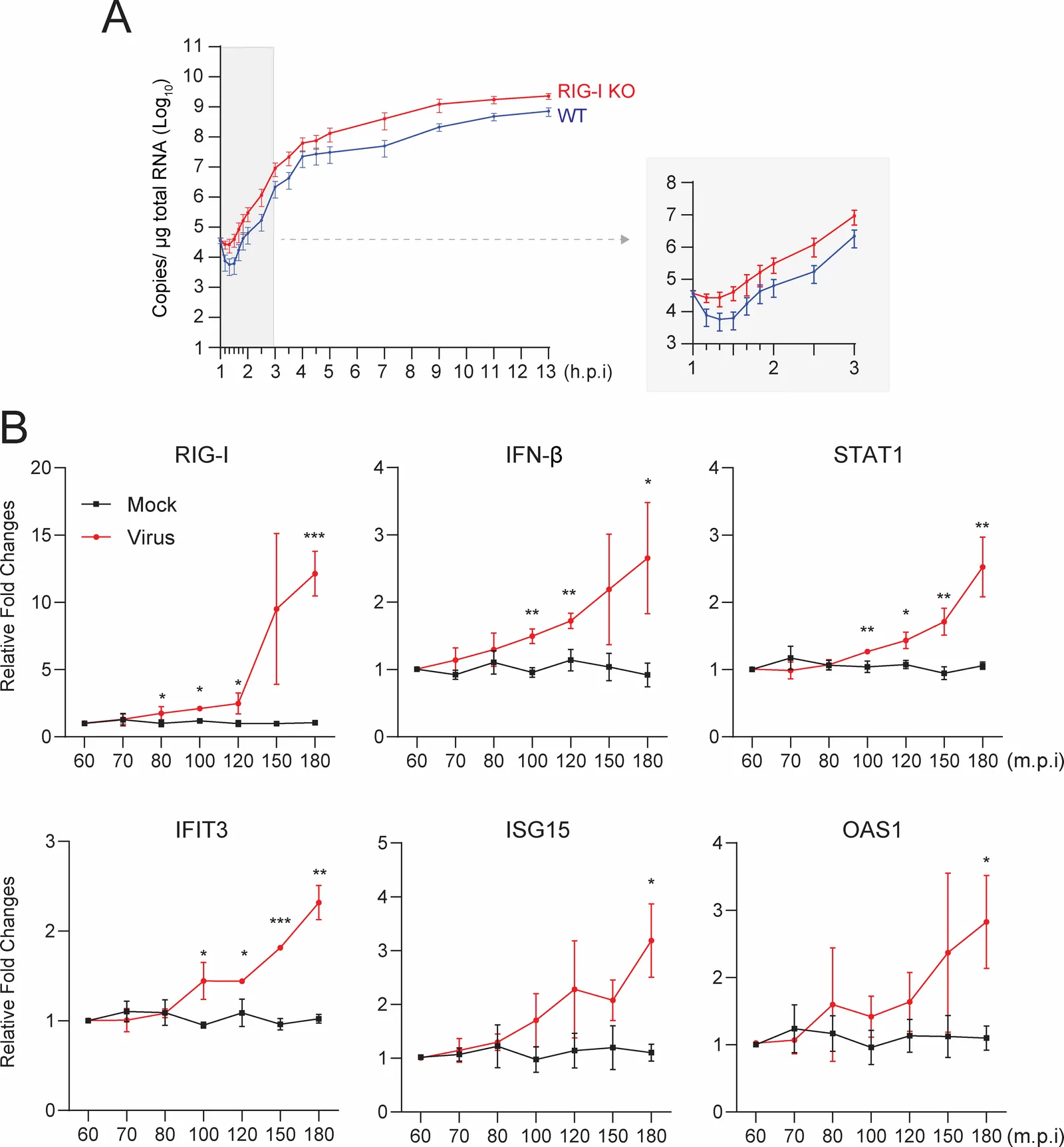
Early influenza vRNA decline precedes strong IFN/ISG transcript induction and is only partially attenuated in RIG-I-deficient cells. (**A**) Strand-specific quantification of NP vRNA in wild-type (WT) and RIG-I-deficient (RIG-I KO) A549 cells following infection with A/WSN/1933 (H1N1) at an MOI of 10. vRNA abundance (log_10_ copies per µg total RNA) was measured at the indicated time points using the Flu-Stranded CRISPR-Cas12a assay. The left subpanel shows the full infection time course (1–13 hpi), and the right subpanel shows an expanded view of the early phase (1–3 hpi). (**B**) Time-course analysis of representative innate immune and interferon-stimulated transcripts in WT A549 cells infected with A/WSN/1933 (H1N1) at an MOI of 10. RIG-I, IFNB1, STAT1, OAS1, IFIT3, and ISG15 transcript levels were measured by RT-qPCR, normalized to GAPDH, and expressed as relative fold change. Expression at the 60 mpi baseline was set to 1 for each gene. Data represent mean ± SD from three independent biological experiments. Statistical comparisons were performed between mock and infected cells at matched time points using a two-tailed Student’s *t*-test; *P < 0.05, **P < 0.01, and ***P < 0.001.

To determine whether host innate RNA sensing initiates the early influenza vRNA decline, we examined viral RNA dynamics in wild-type and RIG-I-deficient A549 cells. RIG-I knockout efficiency was confirmed by immunoblotting and is shown in Fig. S6. We then compared NP vRNA kinetics after A/WSN/1933 infection at MOI 10. In wild-type cells, NP vRNA showed a reproducible early decline during the first 1–2 hpi, followed by recovery and progressive accumulation at later time points (Fig. 6A). A similar early decline was also observed in RIG-I knockout cells, indicating that RIG-I is not essential for initiating the early vRNA loss. However, the magnitude of the early decline was partially attenuated in RIG-I-deficient cells, and subsequent vRNA accumulation was higher than in wild-type cells. These data indicate that RIG-I-associated pathways modulate early viral RNA dynamics and restrict later genome amplification, but they are not the sole driver of the initial vRNA decline.

Because RIG-I signaling operates upstream of canonical IFN/ISG induction, we next asked whether the timing of innate immune gene induction was consistent with a causal contribution of IFN/ISG effectors to the early NP vRNA decline. A549 cells were infected under matched conditions, and the expression of representative innate immune and interferon-stimulated transcripts was measured between 60 and 180 mpi (Fig. 6B). RIG-I transcript levels increased progressively over this period, becoming substantial only at later time points. IFNB1, STAT1, IFIT3, ISG15, and OAS1 were similarly upregulated following infection; however, their induction during the window of early vRNA decline was modest, and more pronounced increases were observed only after vRNA levels had already reached their minimum and begun to recover. Since transcriptional upregulation must precede protein synthesis and the deployment of antiviral effector functions, this temporal pattern argues against newly induced ISG activity as the initiating cause of the early vRNA decrease.

To confirm that the delayed ISG induction described above reflects a genuine antiviral response to productive infection, rather than an artifact of soluble interferon, cytokines, or immunostimulatory contaminants carried over in the viral inoculum, we compared cells exposed to live virus, UV-inactivated virus, virus-depleted stock medium, or mock inoculum (Fig. S7). Only live virus induced ISG15 and OAS1 expression, with modest upregulation at 2 h that became substantially stronger by 4 hpi (Fig. S7A); UV-inactivated virus and virus-depleted medium remained near baseline at both time points. This selectivity was mirrored by infectivity: plaque assay confirmed that live virus retained infectious titer, whereas UV-inactivated virus and virus-depleted medium yielded no detectable plaques (Fig. S7B). The induction of ISG15 and OAS1 therefore tracked with the presence of replication-competent virus, rather than with exposure to the inoculum itself, indicating that productive infection, rather than a soluble or particle-independent component of the viral stock, is required to elicit this early innate immune response. This control confirms that the delayed ISG kinetics reported above reflect a genuine consequence of infection, reinforcing that early vRNA decline precedes the establishment of a productive-infection-triggered IFN/ISG response.

### Early vRNA loss maps to the cytoplasmic incoming-genome pool, whereas importin-β-dependent nuclear entry is required for recovery

Because the timing of innate transcript induction and the RIG-I knockout results argued against *de novo* canonical IFN/ISG activity as the initiating driver of early vRNA loss, we next asked whether this loss arises from the cytoplasmic pool of incoming genomes and whether subsequent nuclear import is required for recovery. To address this, we used importazole, a pharmacological inhibitor of importin-β-dependent nuclear transport (32). Because importin-β also mediates nuclear import of host transcription factors, importazole was used here only as a nuclear-import perturbation tool, not as a pathway-specific inhibitor of viral replication or innate immune signaling (32, 33).

**Fig 7:**
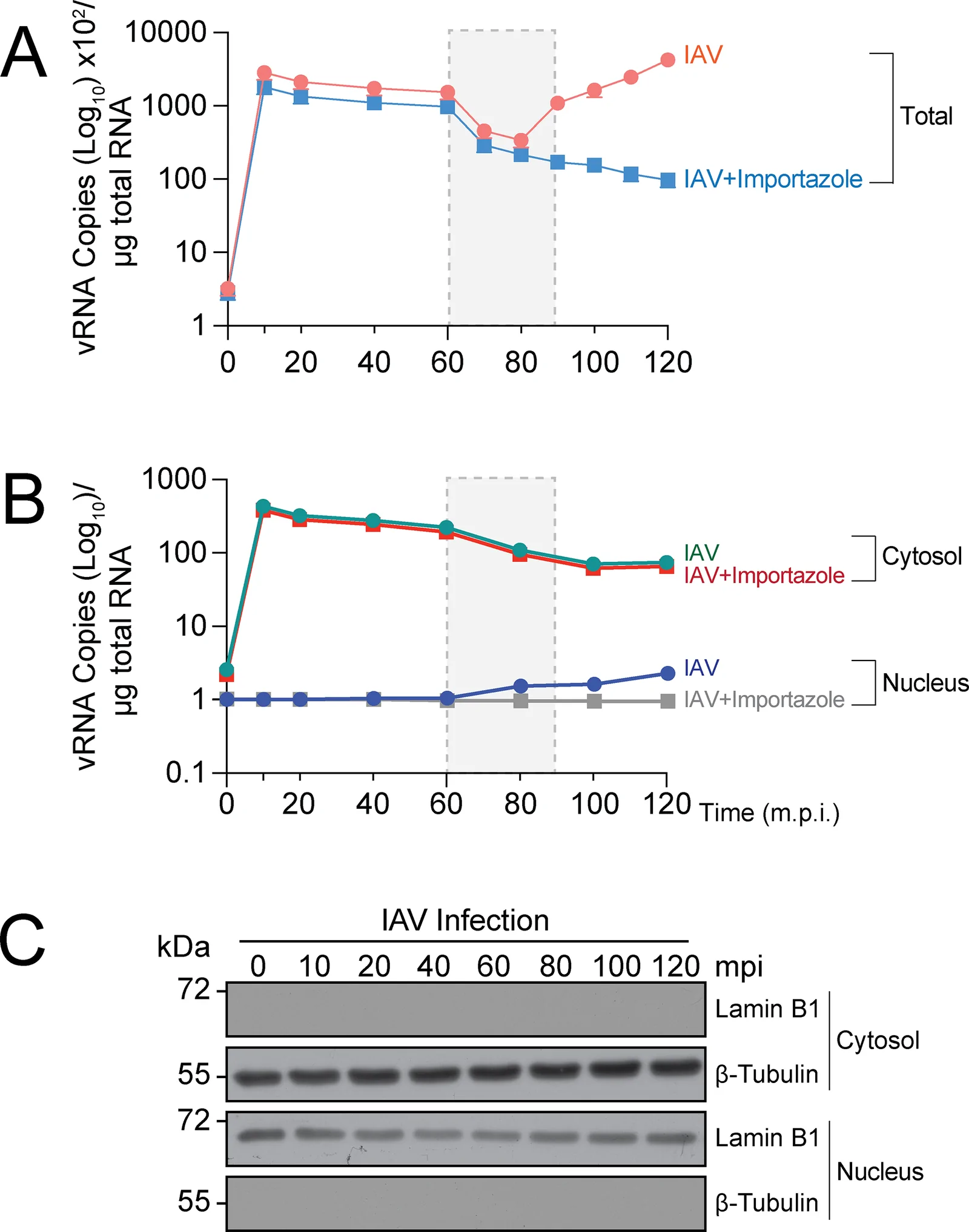
Early vRNA decline is retained in the cytoplasmic fraction, whereas vRNA recovery is associated with importin-β-dependent nuclear accumulation. (**A**) A549 cells were pretreated with 40 µM importazole or DMSO (vehicle control) for 1 h before infection with influenza A/WSN/1933 (H1N1) at an MOI of 10. Virus was added at time 0 without washing to capture total cell-associated input vRNA at inoculation; all subsequent samples were collected after washing to reflect post-entry vRNA dynamics. Cells were maintained in medium containing the respective treatment throughout, and total NP vRNA was quantified at the indicated time points by Flu-Stranded CRISPR-Cas12a. RNA abundance is expressed as log_10_ copies per µg total RNA. The shaded region (60–90 mpi) indicates the interval encompassing the vRNA decline and the onset of divergence between vehicle- and importazole-treated cells. (**B**) Subcellular distribution of NP vRNA following fractionation into cytoplasmic and nuclear compartments across the same time course. DMSO-treated infected cells are shown as green and blue circles, and importazole-treated infected cells are shown as red and gray squares. The shaded region corresponds to the interval highlighted in panel **A**. (**C**) Immunoblot validation of fraction purity using β-tubulin as a cytosolic marker and Lamin B1 as a nuclear marker. Data represent the mean ± SD of three independent biological replicates.

A549 cells were pretreated with importazole for 1 h before infection with A/WSN/1933 at an MOI of 10, and viral RNA species were quantified from 0 to 120 mpi using the Flu-Stranded CRISPR-Cas12a platform (Fig. 7A). Total vRNA increased rapidly within the first 10 min, consistent with efficient entry and detection of incoming viral genomes, then declined progressively, with a more pronounced reduction between approximately 60–80 min (Fig. 7A), corresponding to the early vRNA reduction phase characterized in Fig. 4. This early decline occurred with similar timing in both vehicle- and importazole-treated cells, indicating that the initial vRNA loss does not require importin-β-dependent nuclear import (Fig. 7A). In contrast, vehicle-treated cells showed vRNA recovery from approximately 90 min onward, accompanied by increases in cRNA and mRNA, consistent with initiation of productive viral RNA synthesis (Fig. 7A; Fig. S8A). Importazole abolished this recovery phase, with vRNA, cRNA, and mRNA remaining near post-decline levels throughout the observation period (Fig. S8A). These data indicate that nuclear import is required for recovery and productive amplification, but not for the initial early vRNA decrease.

To define the compartmental origin of these dynamics, we performed subcellular fractionation across the same synchronized 0 to 120 min time course in parallel with importazole treatment, with fractionation quality validated at each time point using compartment-specific protein markers (Fig. 7B and C). In the cytoplasmic fraction, vRNA was readily detectable early after infection, increased transiently at approximately 10 mpi, consistent with cytosolic release of vRNPs during endosomal escape, before declining gradually through 120 min (Fig. 7B). Cytoplasmic vRNA kinetics were highly similar in untreated and importazole-treated cells throughout the time course, indicating that the early decrease in total vRNA is accounted for by loss from the cytoplasmic vRNA pool and is independent of nuclear import. In contrast, nuclear vRNA displayed a temporally delayed and condition-dependent pattern. In untreated cells, nuclear vRNA remained near background during the first 60 min, while cytoplasmic vRNA was already declining, before increasing progressively from approximately 80 to 120 min, coincident with the onset of productive RNA synthesis (Fig. 7B). In importazole-treated cells, nuclear vRNA remained at or near background throughout the entire observation period, consistent with effective inhibition of importin-β-dependent nuclear accumulation of incoming vRNPs.

Because importazole broadly affects importin-β-dependent nuclear transport, we also examined its impact on innate transcriptional outputs. During infection, importazole attenuated induction of RIG-I, IFNB1, STAT1, OAS1, ISG15, and related transcripts (Fig. S8B). In addition, poly(I:C) stimulation experiments showed that importazole strongly reduced poly(I:C)-induced RIG-I, IFNB1, ISG15, and OAS1 expression in the absence of viral replication (Fig. S9). These data confirm that importazole broadly impairs host nuclear signaling outputs and should not be interpreted as a virus-specific inhibitor of innate immune activation. Accordingly, the importazole experiments are used here only to separate early cytoplasmic vRNA attrition from later nuclear-import-dependent recovery. Together, the compartment-resolved data support a two-phase model: incoming vRNA is lost from the cytoplasmic pool during the early post-entry phase, whereas successful nuclear import is required for subsequent vRNA recovery and productive replication.

## DISCUSSION

IAV replicates via a complex cycle involving three structurally related RNA species, vRNA, cRNA, and mRNA, whose strand-specific quantification has been achievable for over two decades through established molecular approaches, but sensitivity and quantitative dynamic range at low RNA abundance remain practical constraints with existing methods (14–17, 34). Building on these established principles, this study develops Flu-Stranded CRISPR-Cas12a, a complementary detection platform that combines strand-specific reverse transcription with a CRISPR-Cas12a-based readout to achieve high sensitivity, broad dynamic range, and compatibility with alternative nucleic-acid detection workflows. The robustness of our assay arises from integrating strand-specific primer tagging with Cas12a collateral cleavage activity, allowing selective detection of structurally similar RNA strands (Fig. 1). This capability was validated rigorously using synthetic RNA templates, demonstrating exclusive fluorescence signal generation for matched guide-template pairs without detectable off-target effects (Fig. 1; Fig. S1). Importantly, this approach maintained high specificity across influenza subtypes and various respiratory viruses, thereby highlighting the potential for broad application in clinical and epidemiological settings (Fig. 1 and 3). In direct benchmarking against established detection methods using identical RNA inputs, the Flu-Stranded CRISPR-Cas12a platform demonstrated reliable quantification down to 10^2^ copies per reaction, extending the lower boundary of the validated quantitative range approximately one to two orders of magnitude beyond both SYBR Green RT-qPCR and TaqMan RT-qPCR (Fig. 2; Fig. S3). Across the broader quantitative range, Cas12a performance was comparable to TaqMan RT-qPCR, and the two platforms are best understood as complementary rather than hierarchically ranked in overall analytical capability. This extended lower detection boundary represents a practically meaningful advantage for applications requiring quantification of early incoming viral genomes, residual RNA following antiviral treatment, or samples near the clinical detection threshold. The platform maintained a quantitative dynamic range spanning approximately 10^2^ to 10^10^ copies across eight orders of magnitude, supporting accurate measurement across the full spectrum of infection from the earliest detectable cytoplasmic genomes through peak replication (Fig. 2; Fig. S3). Therefore, the Flu-stranded CRISPR-Cas12a platform can be applied to track the kinetic changes of IAV RNA species in murine lung tissues, closely linking these changes with significant pulmonary inflammation and consolidation (Fig. 3A through C). It can also detect low-abundance RNA species during the resolution phase to monitor viral clearance and residual replication activity (Fig. 3A through C).

To extend evaluation beyond experimental systems, we applied the Flu-Stranded CRISPR-Cas12a platform to a blinded set of clinical nasopharyngeal swab specimens previously classified by routine diagnostic TaqMan RT-qPCR. In this cohort, the assay demonstrated concordant subtype-level classification across H1N1-positive, H3N2-positive, and influenza-negative samples, consistent with the diagnostic assignments provided by the reference laboratory (Fig. 3D). These findings support the use of Flu-Stranded CRISPR-Cas12a as a strand-resolved and subtype-discriminating detection framework in clinically derived material, with performance evaluated here in terms of classification concordance rather than absolute quantitative equivalence. In this context, the platform may serve as a complementary approach in settings where strand specificity or subtype discrimination is required, or where integration with CRISPR-based detection workflows is advantageous. More broadly, the modular design of the platform may support adaptation to multiplexed CRISPR-based detection strategies for respiratory viruses, although this will require further validation in clinically representative settings. A Fast-Flu: RT-RPA-CRISPR/Cas12a system has been recently developed to detect influenza B virus (35); therefore, there is potential to incorporate multiple CRISPR/Cas12a systems for the diagnosis of clinical respiratory viral infections.

In addition to precisely measuring IAV RNA species under low-input conditions, the Flu-stranded CRISPR-Cas12a platform allows for sensitive detection of their early kinetic changes following IAV infection (Fig. 4; Fig. S4 and S5). These kinetics align well with the known replication-transcription program of influenza. RdRp bound on the incoming vRNP is principally a transcriptional machine at first. It uses the host-derived capped RNA primers (cap-snatching) to synthesize viral mRNAs immediately upon nuclear entry of the vRNP (36). However, the same incoming RdRp complex does not effectively initiate genome replication (36). Previous studies using strand-specific RNA sequencing have shown that influenza’s incoming RdRps are essentially restricted to mRNA synthesis, and that *de novo* generation of cRNA requires newly synthesized polymerase proteins (8). Consistently, our findings showed that mRNA levels rise quickly (as the virus immediately begins transcription), while cRNA remains low during the first hpi. Only after a threshold of new polymerase complexes and NP accumulates do we see a significant increase in cRNA, followed by secondary vRNA synthesis (the “rebound” of vRNA). The uncoupling of mRNA and cRNA kinetics in our results underscores the complex regulation of IAV replication, correlated with previous research where vRNA levels did not strictly align with cRNA (8). It appears the switch from transcription to replication is tightly controlled and temporally segregated, which naturally produces different kinetic curves for each RNA species (7, 8, 12). From a biological perspective, the postponement of cRNA and, consequently, vRNA synthesis until the presence of new viral proteins may function to ensure that replication occurs only when there is a sufficient quantity of NP available to encapsulate and stabilize nascent RNAs (37–39). Any cRNA generated during this early interval may remain transiently exposed and therefore susceptible to degradation in the absence of efficient protection (40). Consistent with this framework, the rebound in vRNA coincides with the onset of *de novo* transcription and replication (7, 14). Our strand-specific data capture this transition from early genome reduction to virus-driven RNA production. Notably, live-cell imaging at single-cell resolution independently recapitulated the same dip-and-rebound trajectory of vRNA, providing orthogonal validation of the population-level kinetics observed by strand-specific quantification (Fig. 5). These findings support the existence of a transient early phase of cytoplasmic genome loss prior to the onset of productive replication.

This early loss of incoming vRNA raises the possibility that host surveillance mechanisms act on viral genomes during cytoplasmic transit. However, multiple independent observations argue that the early decline is not driven by canonical interferon signaling. The persistence of the early decline in RIG-I-deficient A549 cells, together with its partial attenuation, supports a modulatory rather than essential role for RIG-I in early vRNA dynamics, contributing partially to the initial reduction phase and more substantially to limiting downstream replication (Fig. 6A). Consistent with this, IFNB1 and ISG transcripts remained only weakly induced during the early vRNA decline, with stronger induction emerging only at later time points, in agreement with the established requirement for productive viral RNA synthesis and nuclear export upstream of robust interferon responses (41–43). The limited early ISG induction is therefore more consistent with low-level sensing than with functional antiviral effector activity, which would require translation and activation on timescales incompatible with the observed rapid vRNA loss (44, 45). In addition, UV-inactivated virus and virus-depleted stock medium failed to elicit detectable IFNB1 or ISG responses (Fig. S7), arguing against a major contribution from inoculum-derived immunostimulatory factors.

A plausible contributor to the early decline is passive clearance of non-productive input particles. Viral inocula are inherently heterogeneous and include non-infectious virions and defective interfering particles that contribute to initial cell-associated vRNA but fail to establish productive infection. Such genomes are likely routed to endolysosomal degradation during entry and intracellular trafficking (46, 47), consistent with established influenza entry kinetics in which viral fusion and endosomal escape occur before efficient nuclear accumulation of incoming vRNPs, creating an early cytoplasmic transit window during which non-productive genomes may be selectively lost (48). Because defective-particle content was not directly quantified in the present study, we cannot determine the extent to which this mechanism accounts for the observed decline, and we present it as a plausible rather than established contributor.

While the timing of this early decline appears uncoupled from *de novo* interferon signaling, its magnitude is nevertheless influenced by the pre-existing antiviral state of the cell. In IFN-deficient Vero cells, the early vRNA decline is attenuated relative to IFN-competent cells, whereas exogenous IFN-α or IFN-β pretreatment prior to infection restores stronger restriction and reduces subsequent vRNA accumulation (Fig. S10). These findings indicate that interferon signaling is not required to initiate early genome loss but can modulate its extent under conditions of prior antiviral priming. These observations are consistent with a model in which constitutively active or pre-existing RNA surveillance pathways contribute to early genome loss independently of new protein synthesis. Mechanistically compatible candidates include Regnase-1, which mediates stem-loop-dependent cytoplasmic RNA decay and is enhanced by interferon (49, 50), and the ZAP-KHNYN axis, which restricts CpG-enriched viral RNA at baseline expression levels and is further amplified by interferon signaling (51, 52). Defining their relative contributions will require targeted perturbation in human epithelial contexts.

Subcellular fractionation and importazole experiments further support a role for importin-β-dependent nuclear import as a transition point separating cytoplasmic attrition from productive amplification. Cytoplasmic vRNA declined with indistinguishable kinetics in control and importazole-treated cells, whereas nuclear vRNA accumulated from approximately 80 min onward in untreated cells but remained at background level under importazole (Fig. 7B). Importazole also abolished the rebound in total vRNA, cRNA, and mRNA (Fig. S8A), indicating that importin-β-mediated nuclear entry is required for productive genome amplification. These observations support a model in which early vRNA loss reflects depletion of cytoplasmic incoming genomes prior to nuclear import, whereas recovery depends on successful nuclear translocation. Early infection can therefore be viewed as a kinetic competition in which incoming vRNPs must reach the nucleus before they are eliminated, consistent with quantitative models emphasizing cytosolic loss during transit (33, 53–55).

Importazole attenuated antiviral gene induction during infection (Fig. S8B). However, poly(I:C) stimulation in the absence of infection produced a similar attenuation of innate gene induction (Fig. S9), indicating that this effect was not virus-specific, but was consistent with broader inhibition of importin-dependent host transcriptional activation. Because this control does not extend to vRNP nuclear import itself, our conclusion regarding the requirement for nuclear entry does not rely on these transcriptional data, but rests on the compartment-resolved vRNA kinetics described above, in which cytoplasmic attrition was preserved under importazole treatment while nuclear vRNA accumulation and subsequent recovery were not.

Taken together, these data do not resolve a single dominant mechanism for the early vRNA loss. Instead, the observed decline most likely reflects a composite of passive clearance of non-productive particles, unsuccessful uncoating or trafficking trajectories, and constitutive cytoplasmic restriction acting prior to nuclear protection being established. Resolving these relative contributions will require viral stocks with defined particle composition, targeted perturbation of endosomal acidification and trafficking, and single-particle approaches that link entry outcomes to strand-specific RNA trajectories.

Several limitations of this study should be acknowledged. Because importazole broadly inhibits importin-β-dependent transport, it affects both vRNP nuclear import and host transcription factor trafficking, which limits mechanistic attribution based solely on transcriptional readouts alone; this limitation is mitigated by our reliance on compartment-resolved vRNA measurements, rather than transcriptional data, to define the cytoplasmic to nuclear transition. The Vero cell experiments provide supportive evidence that interferon priming can influence vRNA accumulation, but they do not substitute for direct genetic perturbation of interferon signaling in human epithelial cells; IFNAR-deficient A549 models would provide stronger causal evidence in future work. The present kinetic analyses were limited to segments 5 (NP) and 6 (NA), and the temporal patterns described here should not be assumed to generalize uniformly across all eight viral segments without direct testing; the Flu-Stranded CRISPR-Cas12a workflow is, however, technically extensible to the remaining segments using the same strand-specific design principles, providing a framework for future segment-resolved analyses. In addition, digital PCR benchmarking was not performed in this study and would further strengthen analytical validation of the copy-number estimates reported here. Finally, these experiments were conducted using A/WSN/1933 at high MOI; extension to diverse strains, lower-MOI conditions, and viral stocks with defined defective particle content will be important for establishing the generality of the model.

### Conclusions

By applying a strand-specific CRISPR-Cas12a platform with high analytical sensitivity and temporal resolution, we resolved the kinetics of influenza A virus vRNA, cRNA, and mRNA across early and late stages of infection. The platform captured the expected transition from early transcription to subsequent genome replication and revealed a reproducible early decline in incoming vRNA during the post-entry phase. Genetic, kinetic, and compartment-resolved analyses indicate that this early decline is not initiated by *de novo* canonical IFN/ISG activity under the tested conditions, but instead reflects a cytoplasmic attrition phase affecting incoming genomes. RIG-I-associated pathways and the pre-existing antiviral state of the cell can modulate this process, whereas importin-β-dependent nuclear import is required for subsequent vRNA recovery and productive RNA synthesis. In animal tissues and clinical specimens, the platform supported sensitive strand-resolved and subtype-discriminating detection, including reliable quantification within low-copy regimes near the lower limit of validated RT-qPCR performance, complementing conventional RT-qPCR-based approaches. This work establishes Flu-Stranded CRISPR-Cas12a as a useful framework for monitoring influenza RNA species and provides a more refined view of the early post-entry bottleneck preceding productive nuclear replication.

## MATERIALS AND METHODS

### Cells and viruses

Madin–Darby canine kidney (MDCK; RRID:CVCL_0422, ATCC CCL-34), human lung adenocarcinoma epithelial (A549; RRID:CVCL_0023, ATCC CCL-185), human embryonic kidney 293T (HEK293T; RRID:CVCL_0063, ATCC CRL-3216), and African green monkey kidney (Vero; RRID:CVCL_0059, ATCC CCL-81) cell lines were obtained from the American Type Culture Collection (ATCC, Manassas, VA, USA). Human RIG-I knockout (RIG-I-KO) A549 cells (A549 KO-RIG-I; InvivoGen a549d-korigi) were obtained commercially from InvivoGen (San Diego, CA, USA). All cell lines were maintained under standard culture conditions and routinely tested to confirm they were free of mycoplasma contamination. MDCK cells were cultured in Dulbecco’s modified Eagle medium (DMEM; Gibco, USA) supplemented with 10% fetal bovine serum (FBS), 2 mM L-glutamine, 0.1 mM non-essential amino acids, 100 U/mL penicillin, and 100 µg/mL streptomycin. A549, HEK293T, and Vero cells were maintained in DMEM containing 10% FBS and 1% penicillin-streptomycin. All cells were incubated at 37°C in a humidified atmosphere containing 5% CO_2_. For viral infection, cells were inoculated with influenza A/WSN/1933 (H1N1) at the indicated MOI in serum-free DMEM supplemented with 2 μg/mL *N-p*-tosyl-L-phenylalanine chloromethyl ketone (TPCK)-treated trypsin (Sigma-Aldrich, USA). After 1 h of viral adsorption at 37°C, cells were washed twice with phosphate-buffered saline (PBS), maintained in serum-free DMEM containing 1 μg/mL TPCK-treated trypsin, and collected at the indicated time points. Influenza A virus subtype H1N1 (strain A/WSN/1933 [H1N1]) and H3N2 (strain A/Hong Kong/1/1968), as well as influenza B virus (strain B/TW/99/07), enterovirus A71 (EV-A71), enterovirus D68 (EV-D68), and human coronaviruses HCoV-229E and HCoV-OC43 were obtained from the Research Center of Emerging Viral Infection at Chang Gung University, Taiwan.

### *In vitro* transcription of RNA standards

To generate strand-specific RNA standards, template DNA encoding influenza A virus NP and NA segments were PCR-amplified from pPolI-derived plasmids using forward primers containing a T7 promoter sequence (5′-TAATACGACTCACTATAGGG-3′) at the 5′ end. Primer sequences used to generate T7 promoter-containing templates are listed in Table S3. PCR products were purified using the QIAquick PCR Purification Kit (Qiagen, Hilden, Germany). *In vitro* transcription was performed using the RiboMAX Large Scale RNA Production System-T7 (Promega, Madison, WI) at 37°C for 4 h, according to the manufacturer’s instructions. Residual DNA templates were digested with RQ1 DNase I (Promega) at 37°C for 15 min, and the transcribed RNA was subsequently purified using the RNeasy Mini Kit (Qiagen). RNA integrity was verified by agarose gel electrophoresis. RNA concentrations were determined spectrophotometrically, and copy numbers were calculated based on transcript length and molecular weight. The resulting RNA transcripts were used to generate standard curves. For cross-reactivity assays, equimolar inputs corresponding to 2 × 10^9^ copies per reaction of each RNA species were used.

### Design and synthesis of Cas12a guide RNAs

Cas12a gRNAs were designed to target conserved internal regions of the NP and NA gene segments of influenza A/WSN/1933 (H1N1). Candidate guide sequences were selected using Benchling and Integrated DNA Technologies (IDT) design tools based on the LbCas12a-compatible 5′-TTTV-3′ PAM and were screened to minimize potential off-target effects and cross-reactivity with non-target viral sequences. For each viral segment, two guide RNAs were selected: one recognizing the PCR amplicons generated from vRNA-specific tagged reverse transcription and one recognizing the PCR amplicons generated from cRNA- or mRNA-specific tagged reverse transcription. Because cRNA and mRNA share identical internal positive-sense coding sequences and differ mainly in their terminal features, Cas12a gRNA recognition alone cannot distinguish these two RNA species. Therefore, strand specificity was achieved through a two-step strategy consisting of strand-specific tagged reverse transcription, followed by tag-dependent PCR amplification, with Cas12a serving as a downstream sequence-specific cleavage readout for the resulting amplicons. All guide RNAs and synthetic RNA targets corresponding to NP and NA vRNA, cRNA, and mRNA were synthesized by IDT (USA). Primer and guide RNA sequences are listed in Tables S4 and S5.

### Strand-specific reverse transcription and quantitative PCR (SYBR Green and TaqMan)

Cells were harvested at the indicated time point. Total RNA from IAV-infected cells was extracted using TRIzol Reagent (Thermo Fisher Scientific Inc., MA, USA), following the manufacturer’s instructions. Strand-specific reverse transcription was performed using gene-specific primers with unique 5′ non-viral tag sequences (5′-GGCCGTCATGGTGGCGAAT, 5′-GCTAGCTTCAGCTAGGCATC, or 5′-CCAGATCGTTCGAGTCGT) to generate cDNA corresponding to vRNA, cRNA, or mRNA, respectively, as listed in Table S4. Strand-specific reverse transcription was performed using tagged primer strategies adapted from previous reports (14), with modified reaction conditions as described below. Briefly, 1.5 μg of total RNA was mixed with 2 μM tagged RT primer and dNTPs in a total volume of 12 μL, heated at 65°C for 10 min, and immediately chilled on ice for 5 min. Reverse transcription was then performed in a final volume of 20 μL by addition of 4 μL of 5× First-Strand Buffer (Invitrogen), 2 μL of 0.1 M dithiothreitol, 1 μL of RNaseOUT Recombinant Ribonuclease Inhibitor (40 U/μL), and 1 μL of SuperScript III reverse transcriptase (200 U/μL; Invitrogen). Reactions were incubated at 50°C for 2 h, followed by enzyme inactivation at 70°C for 15 min. The resulting strand-specific cDNA products were used as input for both Flu-Stranded CRISPR-Cas12a detection and real-time PCR (qPCR) analyses.

Strand-specific cDNA was subsequently quantified by real-time PCR using either SYBR Green or TaqMan chemistry. SYBR Green qPCR was performed using Smart Quant Green Master Mix (Protech, Taipei, Taiwan), whereas TaqMan qPCR was performed using FastStart Universal Probe Master (Rox) (Roche Diagnostics, Basel, Switzerland), on a QuantStudio 3 Real-Time PCR System (Applied Biosystems, Foster City, CA, USA), according to the manufacturers’ instructions. Primers and probes used for detection of vRNA, cRNA, and mRNA are listed in Tables S4, S6, and S7.

For absolute quantification, assay-specific standard curves were generated for each qPCR workflow using 10-fold serial dilutions (10^10^ to 10^0^ copies per reaction) of *in vitro*-transcribed RNA standards corresponding to each viral RNA species. The same IVT RNA standards were processed through the identical strand-specific reverse transcription workflow and used as input for both Flu-Stranded CRISPR-Cas12a and qPCR analyses, ensuring direct comparability between platforms. Absolute copy numbers for each platform were calculated independently using their respective standard curves, and all samples were analyzed within the linear dynamic range of the corresponding assay.

### Flu-Stranded CRISPR-Cas12a assay

The Flu-Stranded CRISPR-Cas12a assay integrates strand-specific reverse transcription, PCR amplification of the resulting cDNA, and LbCas12a-mediated trans-cleavage detection. LbCas12a protein was obtained from New England Biolabs (Ipswich, MA, USA).

### PCR amplification

Strand-specific cDNA was amplified using HiFi DNA polymerase master mix (TOOLS Biotech, Taiwan) with the same cDNA input used for qPCR comparison. PCR cycling conditions were as follows: initial denaturation at 94°C for 3 min, followed by 30 cycles of 94°C for 30 s, 55°C for 30 s, and 72°C for 40 s, with a final extension at 72°C for 5 min. Primers used for amplification are listed in Table S4.

### CRISPR-Cas12a detection assay

The LbCas12a-gRNA ribonucleoprotein complex was preassembled in a 20 µL mixture containing 75 nM LbCas12a, 150 nM gRNA, and 1× NEBuffer 3.1 for 30 min at 37°C prior to use. Fluorescence detection reactions were performed in a final volume of 50 µL containing the preassembled complex (final concentrations: 30 nM LbCas12a and 60 nM gRNA), 500 nM fluorescent single-stranded DNA reporter (5′-6-FAM-TTATT-BHQ1-3′; Integrated DNA Technologies), 1× NEBuffer 3.1, and 3 µL of PCR product. Reactions were assembled in black 96-well flat-bottom plates (Corning) and incubated at 37°C for 30–60 min. Fluorescence was monitored in real time at 60 s intervals using a Synergy H1 plate reader (BioTek, USA) with excitation and emission wavelengths of 485 and 535 nm, respectively. Reactions lacking target DNA, Cas12a protein, or matched gRNA were included as negative controls to assess background and assay specificity. Fluorescence data were collected using Gen5 software (BioTek, USA). gRNAs are listed in Table S5.

### Absolute quantification

For absolute quantification, assay-specific standard curves were generated using 10-fold serial dilutions (10^10^ to 10^0^ copies per reaction) of *in vitro*-transcribed RNA standards corresponding to each viral RNA species. These standards were processed through the same strand-specific reverse transcription, PCR amplification, and Flu-Stranded CRISPR-Cas12a workflow as experimental samples. Fluorescence signals were quantified as the net increase (ΔF), calculated as the fluorescence intensity at 30 min minus the baseline signal at 0 min for each individual reaction. This baseline correction removes background fluorescence from reaction components and accounts for well-to-well variation in initial signal. The 30 min time point was selected for quantification as it provided an optimal balance between signal intensity and linearity within the dynamic range of the assay. These background-corrected fluorescence signals (ΔF) were converted to RNA copy numbers by interpolation using standard curves generated from matched *in vitro*-transcribed RNA standards. No-template and no-target controls were included on each plate to define baseline signal and assess nonspecific background fluorescence. All measurements were performed using independent biological replicates, and data were summarized as mean ± standard deviation (SD).

### Quantification of host immune gene expression by RT-qPCR

Cells were harvested at the indicated time points, and total RNA was extracted using TRIzol Reagent (Thermo Fisher Scientific) according to the manufacturer’s instructions. RNA was then reverse transcribed using random hexamers and M-MLV reverse transcriptase (Thermo Fisher Scientific) to generate cDNA. Quantitative PCR was performed using Smart Quant Green Master Mix (Protech, Taipei, Taiwan) on a QuantStudio 3 Real-Time PCR System (Applied Biosystems). Gene expression levels of RIG-I, IFNB1, STAT1, IFIT3, ISG15, and OAS1 were measured using gene-specific primers listed in Table S8. Relative expression levels were normalized to GAPDH as an internal reference control and calculated using the 2^−ΔΔCt^ method.

### Importazole treatment and subcellular fractionation

A549 cells were pretreated with importazole (40 μM; Sigma-Aldrich) for 1 h at 37°C and 5% CO₂. Cells were then infected with influenza A/WSN/1933 (H1N1) at MOI 10 in serum-free DMEM supplemented with TPCK-treated trypsin and 40 μM importazole at 37°C, and importazole was maintained throughout the time course. The 0 min time point was collected immediately upon inoculum addition without washing, representing total cell-associated input vRNA at inoculation. For all subsequent time points, unbound inoculum was removed by two PBS washes prior to harvesting. At each indicated time point, cells were harvested and cytoplasmic and nuclear fractions were isolated using the TOOLS Cytoplasmic and Nuclear Extraction Kit (TOOLS Biotech, Taiwan) according to the manufacturer’s protocol. Each fraction was divided for protein analysis by immunoblot and RNA extraction using TRIzol Reagent (Invitrogen). Fractionation efficiency was confirmed by immunoblot using anti-α-tubulin as a cytoplasmic marker and anti-lamin B1 as a nuclear marker at each time point (Fig. 7C). For total RNA kinetics, cells were harvested in parallel, and RNA was extracted directly using TRIzol without fractionation.

### Time-lapse live-cell imaging of vRNA dynamics

IAV vRNA dynamics were visualized according to our previous study (31). HEK293T cells were seeded onto glass coverslips or Attofluor cell chambers (Invitrogen) at a density of 8 × 10⁴ cells per well and incubated overnight at 37°C in a humidified atmosphere containing 5% CO_2_. The following day, cells were co-transfected with 1 μg each of pHH-PB2-vMSL (encoding the PB2 segment containing 24× MS2 stem-loop sequences) and pMCP-GFPdN (encoding the MS2 coat protein lacking a nuclear localization signal) using Lipofectamine 2000 (Thermo Fisher Scientific), according to the manufacturer’s protocol. At 20 h post-transfection, cells were washed with serum-free DMEM and infected with A/WSN/1933 (H1N1) virus at an MOI of 10. Infection was carried out in serum-free DMEM supplemented with 200 ng/mL TPCK-treated trypsin for 1 h at 37°C. Following infection, cells were maintained in the same medium for live imaging. Time-lapse fluorescence imaging was performed using a Leica DMi8 inverted microscope equipped with a Tokai Hit stage-top incubation system to maintain stable environmental conditions (37°C, 5% CO_2_, humidified). Images were captured every 20 min for a total duration of 4 h using a 100×/1.49 NA oil immersion objective and an ORCA-Flash 4.0 Digital CMOS camera (model C11440-22CU, Hamamatsu). Green fluorescence signals were acquired under 25% LED illumination with 300 ms exposure time. At each time point, 30 z-stacks were collected at 0.3 μm intervals. Image stacks were processed in ImageJ to generate maximum intensity projections. Quantification of vRNA puncta was performed using the CAPS program, which calculates background-subtracted fluorescence intensities across z-planes using photometric analysis. All imaging conditions were kept constant between experimental replicates to ensure reproducibility.

### Mouse infection model and sample collection

Female BALB/c mice (6–8 weeks old, Jackson Laboratory) were intranasally inoculated with 50 μL of PBS containing 9 × 10^3^ PFU of influenza A/WSN/1933 (H1N1). Mock-infected controls received PBS alone. Lung and tracheal tissues were collected at 4 and 7 dpi. Animals were euthanized by CO_2_ asphyxiation, and tissues were immediately harvested and homogenized in TRIzol Reagent (Invitrogen) for RNA extraction. Homogenates were centrifuged at 500 × *g* for 5 min, and RNA was isolated from the clarified supernatant according to the manufacturer’s instructions. Strand-specific detection of viral RNA species (vRNA, cRNA, and mRNA) was performed using Flu-Stranded CRISPR-Cas12a assays and SYBR Green RT-qPCR as described above.

### Clinical sample collection and RNA extraction

Nasopharyngeal swab specimens were collected from patients admitted to Chang Gung Memorial Hospital during routine clinical testing across the 2024–2025 influenza seasons and stored in viral transport medium. Total RNA was extracted using TRI Reagent (Invitrogen) according to the manufacturer’s instructions. The clinical cohort included 48 H1N1-positive samples, 9 H3N2-positive samples, and 1 influenza-negative control sample, as determined by routine diagnostic TaqMan RT-qPCR performed by the clinical laboratory. Specimens were provided to the research laboratory as a fully blinded cohort, without subtype assignments or Ct values at the time of Flu-Stranded CRISPR-Cas12a analysis. Subtype classification and corresponding diagnostic Ct values were released by the clinical laboratory only after completion of all CRISPR-based analyses. SYBR Green RT-qPCR was performed on the same extracted RNA as an in-laboratory comparator.

### Statistical analysis

All experiments were performed with a minimum of three independent biological replicates. Data are reported as mean  ± SD. Statistical analyses were conducted using GraphPad Prism (v9.0). Two-tailed Student’s *t*-tests were used for pairwise comparisons. Linear regression analyses of standard curves, including slope, intercept, and R² values, were also performed using GraphPad Prism (v9.0). Statistical significance was defined as follows: ns, not significant; * and #, *P* < 0.05; ** and ##, *P* < 0.01; *** and ###, *P* < 0.001; **** and ####, *P* < 0.0001. The specific comparisons denoted by each symbol are indicated in the corresponding figure legends.

## Data Availability

The data that support the findings of this study are available from the corresponding author upon reasonable request.

## SUPPLEMENTAL MATERIAL

Supplemental Material mbio.01789-26-s0001.docx Supplemental figures and tables.

ASM does not own the copyrights to Supplemental Material that may be linked to, or accessed through, an article. The authors have granted ASM a non-exclusive, world-wide license to publish the Supplemental Material files. Please contact the corresponding author directly for reuse.
