# Supplementary material for: Monitoring kinetic changes and restriction of influenza A virus RNA species during infection using a Flu-Stranded CRISPR platform
Source: mBio. 2026 Sep 14;17(10):e01789-26. doi: 10.1128/mbio.01789-26 (PMC13712887; doi:10.1128/mbio.01789-26)
Supplement: Supplementary file 1 [file mbio.01789-26-s0001.docx]

# **Supporting information**

**Monitoring Kinetic Changes and Restriction of Influenza A Virus RNA Species During Infection Using a Flu-Stranded CRISPR Platform**

Anh-Tu Tran^1,2,3^, Wen-Fang Tang^2,3^, Chung-Fan Hsieh^3,4^, Ya-Fang Chiu^1,3,5,6^, Chung-Guei Huang^7^, Kuo-Ming Lee^3,8^, Rei-Lin Kuo^3, 8, 9^, Kuei-Ching Hsiung^3^, Cheng-Hsun Chiu^4^, Jim-Tong Horng^1, 2, 3, 4, 10 *^

^1^Graduate Institute of Biomedical Sciences, Chang Gung University, Taoyuan 333323, Taiwan

^2^Department of Biochemistry and Molecular Biology, College of Medicine, Chang Gung University, Taoyuan 333323, Taiwan

^3^Research Center for Emerging Viral Infections, Chang Gung University, Taoyuan 333323, Taiwan

^4^Molecular Infectious Disease Research Center, Chang Gung Memorial Hospital at Linkou, Taoyuan 333423, Taiwan.

^5^Division of Infectious Diseases, Department of Medicine, Chang Gung Memorial Hospital, New Taipei 236017, Taiwan

^6^Department of Microbiology and Immunology, Chang Gung University, Taoyuan 333323, Taiwan

^7^Department of Laboratory Medicine, Linkou Chang Gung Memorial Hospital, Taoyuan 333423, Taiwan

^8^International Master Degree Program for Molecular Medicine in Emerging Viral Infections, Chang Gung University, Taoyuan 333323, Taiwan

^9^[Department of Medical Biotechnology and Laboratory Science](https://pure.lib.cgu.edu.tw/en/organisations/department-of-medical-biotechnology-and-laboratory-science), Chang Gung University, Taoyuan 333323, Taiwan

^10^Research Center for Chinese Herbal Medicine, College of Human Ecology, Chang Gung University of Science and Technology, Taoyuan City 333323, Taiwan

*Corresponding author

Jim-Tong Horng

jimtong@mail.cgu.edu.tw

**Supplementary Figure S1. Strand specificity and subtype selectivity of Flu-Stranded CRISPR-Cas12a for the NA segment of IAV. (A)** Strand specificity of gRNA-NA-v and gRNA-NA-c/m assessed using equimolar synthetic NA vRNA, cRNA, and mRNA (2 × 10^9^ copies per reaction) in the presence of 1 µg of total RNA from mock-infected cells. Fluorescence was measured at 30 min and is expressed as arbitrary units (AU). **(B, C)** Validation of strand-resolved detection of Flu-Stranded CRISPR-Cas12a assay using synthetic vRNA, cRNA, or mRNA of the NA or NP with gRNA-NA-v **(B)** and gRNA-NA-c/m **(C)**. Reactions without synthetic RNA template served as negative controls. **(D, E)** Pathogen specificity analysis of gRNA-NA-v **(D)** and gRNA-NA-c/m **(E)**, tested against a panel of respiratory viruses including H1N1, H3N2, influenza B, human coronaviruses (229E, OC43), and enteroviruses (EV-A71, EV-D68). Total RNA inputs were normalized to 500 ng per reaction across all viral samples. Data represent the mean ± SD of three independent biological replicates.

**Supplementary Figure S2. Flu-Stranded CRISPR-Cas12a maintains strand specificity in the presence of cellular RNA. (A)** Strand-specific detection of NP vRNA, cRNA, and mRNA in a cellular RNA background. Each synthetic NP RNA species was tested individually across all three strand-specific reverse transcription assays targeting vRNA, cRNA, or mRNA, in the presence of 0, 0.5, or 1 µg of total RNA isolated from uninfected MDCK cells, followed by PCR amplification and Cas12a-mediated fluorescence detection. Fluorescence was observed exclusively for the cognate RNA species and was maintained across all background RNA conditions, whereas noncognate RNA targets, no-RT controls, and reactions lacking synthetic RNA template remained at baseline. **(B)** Strand-specific detection of NA vRNA, cRNA, and mRNA under identical conditions. Each synthetic NA RNA species was assessed across all three strand-specific assays in the presence of increasing amounts of uninfected MDCK total RNA. As observed for NP, fluorescence was detected only from the cognate target strand and was preserved across all background RNA conditions, while noncognate strands and negative controls remained at baseline. Fluorescence was measured at 30 min and is expressed as arbitrary units (AU). Data represent the mean ± SD of three independent biological replicates.

**Supplementary Figure S3. Flu-Stranded CRISPR-Cas12a enables high-sensitivity, strand-specific detection of NA vRNA, cRNA, and mRNA across a broad quantitative range.** A 10-fold serial dilution series of synthetic strand-specific NA vRNA, cRNA, or mRNA standards (10^10^ to 10^0^ copies per reaction) was processed through the complete detection workflow and analyzed by Flu-Stranded CRISPR-Cas12a (**A, B**) or SYBR Green RT-qPCR (**C**). **(A)** Real-time fluorescence signals of the Flu-Stranded CRISPR-Cas12a assay for NA vRNA, cRNA, and mRNA across the indicated RNA input range (10^10^ to 10^0^ copies per reaction). Fluorescence is shown as arbitrary units (AU) over 60 min. (**B**) Standard curves generated from Cas12a fluorescence measured at 30 min, plotted against log_10_ transformed input RNA copy number to evaluate assay linearity and dynamic range. **(C)** Standard curves for SYBR Green RT-qPCR generated using the same strand-specific RNA standards, with Ct values plotted against log_10_ transformed input RNA copy number. In panels B and C, the fitted line represents linear regression calculated using data points within the experimentally validated quantitative range. The validated quantitative ranges were 10^2^ to 10^10^ copies for Flu-Stranded CRISPR-Cas12a and 10^3^ to 10^8^ copies for SYBR Green RT-qPCR. Data points outside the linear response range, defined by signal compression at the upper boundary or fluorescence indistinguishable from background at the lower boundary, were excluded from regression analysis. **(D)** Comparison of NA vRNA, cRNA, and mRNA levels in MDCK cells infected with influenza A/WSN/1933 (H1N1) at an MOI of 0.01, 0.1, or 1. Total RNA was collected at 8 hpi and quantified using the Flu-Stranded CRISPR-Cas12a assay (red bar) or RT-qPCR (gray). Results are expressed as log_10_ RNA copies per μg total RNA. Data represent mean ± SD from three independent experiments.

**Supplementary Figure S4. Comparison of Flu-Stranded CRISPR-Cas12a assay** **and RT-qPCR for detecting the kinetic changes of IAV NA vRNA, cRNA, and mRNA.** NA vRNA, cRNA, and mRNA in MDCK cells infected with influenza A/WSN/1933 (H1N1) at MOI 10 were harvested at 1-13 hpi and analyzed via the SYBR qPCR **(A)** and Flu-Stranded CRISPR-Cas12a assay **(B)**. The left panel shows the full time-course (1-13 hpi); the right panel shows the early-phase kinetics (1-3 hpi) at higher resolution. RNA copy numbers are expressed as log_10_ copies per μg total RNA. Data represent the mean ± SD of three independent biological replicates.

**Supplementary Figure S5. Time-resolved quantification of NP- and NA-derived influenza A virus RNA species in A549 cells by Flu-Stranded CRISPR-Cas12a.** vRNA, cRNA, and mRNA derived from the NP (**A**) and NA (**B**) segments were quantified in A549 cells infected with influenza A/WSN/1933 (H1N1) at MOI 10. Cells were harvested at 1 to 13 hpi, and viral RNA species were analyzed using the Flu-Stranded CRISPR-Cas12a assay. The left panel shows the full time-course (1-13 hpi); the right panel shows the early-phase kinetics (1-3 hpi) at higher resolution. RNA copy numbers are expressed as log_10_ copies per μg total RNA. Data represent the mean ± SD of three independent biological replicates.

**Supplementary Figure S6. Validation of RIG-I knockout efficiency in A549 cells.** Wild-type and RIG-I KO A549 cells were left untreated or stimulated with poly(I:C), and RIG-I protein expression was assessed by immunoblotting. RIG-I was detected and induced by poly(I:C) in WT cells but was not detected in RIG-I KO cells under the conditions tested. GAPDH served as the loading control.

**Supplementary Figure S7. Live virus, but not UV-inactivated virus or virus-depleted stock medium, induces ISG15 and OAS1 expression. (A)** A549 cells were mock treated or exposed to live influenza A/WSN/1933 (H1N1) virus, UV-inactivated virus prepared from the same viral stock, or virus-depleted stock medium. ISG15 and OAS1 transcript levels were measured by RT-qPCR at 0, 2, and 4 h post-exposure, normalized to GAPDH, and expressed as relative fold change. Live virus induced modest ISG15 and OAS1 expression at 2 h and stronger induction at 4 h, whereas UV-inactivated virus and virus-depleted medium remained near baseline under the conditions tested. **(B)** Plaque assay of the corresponding inocula used in **(A)**. Live virus produced detectable plaques, whereas UV-inactivated virus and virus-depleted medium showed no detectable plaque formation under the assay conditions. Data in **(A)** represent mean ± SD from three independent biological experiments. Statistical significance was determined by two-tailed Student’s t-test comparing each condition with mock at the corresponding time point; ns, not significant; *p < 0.05; **p < 0.01; ****p < 0.0001.

**Supplementary Figure S8. Importazole prevents influenza viral RNA recovery and attenuates infection-associated antiviral gene expression. (A)** A549 cells were pretreated with 40 μM importazole or DMSO vehicle control for 1 h at 37 °C, then infected with influenza A/WSN/1933 at an MOI of 10 on ice for 10 min to restrict viral attachment to the cell surface and synchronize infection. After washing with PBS to remove unbound virus, cells were maintained in serum-free DMEM containing importazole and TPCK-treated trypsin. Total RNA was collected at the indicated time points, and NP vRNA, cRNA, and mRNA were quantified using Flu-Stranded CRISPR-Cas12a. **(B)** Relative expression of representative antiviral genes, including RIG-I, IFNB1, ISG15, and OAS1, was measured by RT-qPCR and normalized to GAPDH, and expressed as relative fold change. Expression at 60 mpi was set to 1 for each gene. Data represent mean ± SD from three independent biological replicates. Statistical significance was assessed by two-tailed Student’s t-test. Asterisks (*) indicate comparisons between mock and IAV-infected cells at the corresponding time point; hash symbols (#) indicate comparisons between IAV-infected and IAV+ importazole-treated cells at the corresponding time point. *, # p < 0.05; **, ## p < 0.01; ***, ### p < 0.001; ****, #### p < 0.0001.

**Supplementary Figure S9. Importazole broadly suppresses poly(I:C)-induced innate immune transcription.** A549 cells were mock treated, treated with importazole alone, stimulated with poly(I:C), or stimulated with poly(I:C) in the presence of importazole. Expression of RIG-I **(A)**, IFNB1 **(B)**, ISG15 **(C)**, and OAS1 **(D)** was quantified by RT-qPCR at 0 and 6 h post-treatment, normalized to GAPDH, and displayed as relative fold change. Poly(I:C) induced robust expression of RIG-I, IFNB1, ISG15, and OAS1 at 6 h. Co-treatment with importazole substantially attenuated this induction, whereas importazole alone remained comparable to mock treatment. These data indicate that importazole broadly impairs innate transcriptional outputs and is not specific to influenza vRNP nuclear import. Data represent mean ± SD from three independent biological experiments. Statistical comparisons were performed using a two-tailed Student’s t-test; brackets indicate the comparisons shown; ns, not significant; *p < 0.05; **p < 0.01; ***p < 0.001**.**

**Supplementary Figure S10. Exogenous type I interferon pretreatment suppresses IAV vRNA accumulation in Vero cells. (A-C)** NP vRNA, cRNA, and mRNA were quantified by Flu-Stranded CRISPR-Cas12a in Vero cells that were untreated **(A)** or pretreated with recombinant human IFN-α **(B)** or IFN-β **(C)** at 50 IU/mL for 16 h prior to infection, with treatment maintained through 1 hpi, followed by influenza A/WSN/1933 (H1N1) infection at MOI 10. RNA abundance was measured at the indicated time points post-infection. **(D)** Expression levels of antiviral host genes (RIG-I, STAT1, OAS1, ISG15, RNase L, and IFN-β) in mock-infected (green), virus-infected (red), and IFN-β-pretreated virus-infected (purple) Vero cells at the indicated time points, measured by RT-qPCR and normalized to GAPDH mRNA. Expression at 1 hpi was set to 1. Data represent the mean ± SD of three independent experiments. Statistical differences between conditions were assessed by a two-tailed Student’s t-test; **p < 0.01; *** p < 0.001.

**Supplementary Table S1. Intensities of PB2-vMSL signals in HEK293T(MCP-GFPdN) cells infected by IAV**

| **Time^a^**  **Minutes** | **Number of signals^b^** | **Sum of intensities^c^** | **Intensities of individual signal in the cell shown in figure 5**  **[Arbitrary Unit (AU)]^c^** |
| --- | --- | --- | --- |
| 0 | 5 | 226455029 | 54826917; 49269247; 46451925; 43144452; 32762488 |
| 10 | 4 | 177979240 | 53815515; 45534862; 45436046; 33192817 |
| 20 | 3 | 136414549 | 50901534; 43753894; 41759122 |
| 30 | 3 | 129970942 | 46975163; 43609985; 39385794 |
| 40 | 3 | 132544312 | 46103506; 43659971; 42780835 |
| 50 | 4 | 162990510 | 47026443; 45224430; 35158321; 35581317 |
| 60 | 4 | 196682464 | 50349230; 52045553; 50183715; 44103966 |
| 70 | 6 | 249573615 | 54209290; 51446795; 44460735; 44498838; 30673473; 24284483 |
| 80 | 7 | 290633237 | 57950379; 47378415; 46558633; 41728971; 38762869; 32621090; 25632880 |
| 90 | 10 | 323265283 | 57911423; 47735194; 44408268; 41320209; 39568984; 35366568; 16721389; 14770591; 12865733; 12596923 |
| 100 | 13 | 366292607 | 53048349; 47944804; 44831285; 42835837; 37429982; 34801563; 18656522; 16832994; 16310352; 15132027; 13131536; 11373262; 13964094 |
| 110 | 15 | 419920410 | 55624216; 48623795; 47063506; 44571690; 34836083; 29772676; 27579511; 26986413; 16836577; 16594973; 15903065; 15550591; 14658342; 14277275; 11041696 |
| 120 | 17 | 479106291 | 54463399; 47821158; 45209122; 41493662; 35896379; 31594559; 30966372; 29089532; 27120014; 25874084; 24073218; 16311177; 15482661; 14098003; 13915425; 13791081; 11906444 |
| 130 | 18 | 517850293 | 56900587; 49440949; 48174370; 42813336; 40926856; 38870040; 31759836; 25135869; 25405092; 24837245; 23380661; 14895886; 15523711; 13267090; 10510374; 19133004; 18566159; 18309229 |
| 140 | 20 | 545126097 | 52687474; 48857615; 46363546; 42320197; 38149320; 32593567; 32288708; 29959139; 27851686; 25284407; 25136299; 23155636; 15783402; 15742946; 15726526; 14782511; 14683940; 14030961; 11596779; 18131438 |
| 150 | 22 | 565220766 | 58209145; 52262381; 47783655; 43593448; 41716189; 33217998; 30163126; 28338675; 25508472; 25543668; 23684175; 16874509; 16333151; 16216015; 15831611; 16376118; 16363907; 15310604; 14878484; 13593535; 13421901 |
| 160 | 27 | 603586967 | 53207763; 42382418; 36589419; 31376543; 28660834; 25736184; 25655321; 24446347; 23058609; 21346358; 19435271; 20012359; 36098903; 17051044; 16931531; 16043935; 15540987; 14488523; 14030388; 13882821; 13139546; 12493420; 11571475; 11221175; 11089777; 20377029; 27718988 |
| 170 | 28 | 645420989 | 53679665; 42399434; 37266859; 31458581; 28992119; 26748855; 25828559; 24535067; 23569366; 22121880; 17196743; 20229123; 36321306; 16620231; 16465639; 16168224; 17194859; 15052506; 14932794; 13368321; 12662923; 12764266; 12289131; 12352877; 12166595; 22670683; 36591802; 23772582 |
| 180 | 29 | 673750623 | 54492644; 43542048; 37950104; 32480112; 29461604; 27089499; 27366716; 24767093; 23638888; 22641539; 18831523; 21176387; 37135378; 17785737; 17644630; 16373493; 15478379; 15253293; 15181640; 15121851; 13206784; 12854571; 12374433; 12740704; 12227578; 23847437; 38856449; 26062168; 8167942 |
| 190 | 30 | 702999871 | 54624358; 43763109; 39805349; 34026007; 29559455; 27380869; 27486075; 25136865; 24514935; 23021398; 20550471; 21619985; 39009059; 19292047; 18230376; 16866822; 15700764; 15413518; 15226963; 15200118; 15087438; 13066747; 12839099; 12866370; 12615524; 24347559; 42563808; 28559268; 9595674; 5029842 |
| 200 | 32 | 728078356 | 57827083; 48384718; 42274818; 34709252; 30242700; 28153395; 28169320; 27282686; 26027815; 25478550; 8818605; 23016959; 39606759; 18493781; 16364169; 17696312; 16714155; 16005204; 14788893; 14625573; 14523758; 13351846; 12974921; 12985692; 12620888; 24598436; 43620445; 30623202; 9735466; 9170793; 4884568; 4307593 |
| 210 | 34 | 809979937 | 58012930; 47428296; 43456398; 35555706; 30763396; 29764024; 29481257; 27808592; 26655671; 26161795; 29785603; 23700204; 46246507; 22204601; 21600722; 20114970; 21399148; 14787802; 10931659; 10917362; 19446382; 13352527; 13063806; 13073946; 12904185; 24852015; 45114872; 31012705; 25033982; 9457187; 8840315; 8788382; 4145278; 4117714 |
| 220 | 34 | 811748170 | 57063360; 49539508; 48027972; 35267327; 31429966; 30189797; 31351630; 28965931; 27711060; 28797492; 25384712; 28021999; 40883796; 18240337; 16709335; 18823672; 17397400; 16688449; 15472138; 15308818; 15207003; 14035091; 13522344; 13423949; 12910823; 25098256; 45805458; 33219663; 10418711; 9539622; 9251865; 9082877; 9572372; 9385438 |
| 230 | 36 | 866961646 | 58133548; 51121008; 49490861; 37536123; 33538546; 31873042; 33481257; 29402069; 28769702; 29894790; 26067957; 30733997; 44880017; 20521356; 20917477; 19506917; 20197484; 13758486; 17401808; 13210349; 21161646; 14852654; 13596346; 20876447; 19587402; 31064059; 47430752; 11628892; 10057329; 9962281; 9182450; 10140432; 9935110; 9766122; 8691225; 8591704 |

^a^ HEK293T cells were co-transfected with plasmids expressing MCP‑GFPdN and the PB2‑vMSL reporter for 20 hours, and subsequently infected with influenza A/WSN/1933 (H1N1) for live-cell imaging at 10-minute intervals from 60 to 300 m.p.i..

^b^ Total number containing vRNA puncta in cells and ^c^ their intensity of signals in Arbitrary Unit (A.U.) were determined by CAPS (1,2) .

**Supplementary Table S2. Intensities of PB2-vMSL signals in HEK293T(MCP-GFPdN) cells**

| **Time^a^**  **Minutes** | **Number of signals^b^** | **Sum of intensities^c^** | **Intensities of individual signal in the cell shown in figure 5**  **[Arbitrary Unit (AU)]^c^** |
| --- | --- | --- | --- |
| 60 | 5 | 141249521 | 39970679; 29045092; 25781825; 21387311; 25064614 |
| 70 | 5 | 141249738 | 39970718; 29045132; 25781871; 21387356; 25064661 |
| 80 | 5 | 141249953 | 39970757; 29045172; 25781916; 21387402; 25064707 |
| 90 | 5 | 141250166 | 39970795; 29045212; 25781961; 21387446; 25064752 |
| 100 | 5 | 141250377 | 39970833; 29045251; 25782005; 21387491; 25064797 |
| 110 | 5 | 141250586 | 39970871; 29045289; 25782049; 21387535; 25064842 |
| 120 | 5 | 141250793 | 39970908; 29045328; 25782093; 21387579; 25064886 |
| 130 | 5 | 141250998 | 39970944; 29045366; 25782136; 21387622; 25064930 |
| 140 | 5 | 141251201 | 39970981; 29045403; 25782179; 21387665; 25064974 |
| 150 | 5 | 141251402 | 39971017; 29045440; 25782221; 21387707; 25065017 |
| 160 | 5 | 141251601 | 39971052; 29045477; 25782263; 21387750; 25065059 |
| 170 | 5 | 141251798 | 39971087; 29045513; 25782305; 21387791; 25065102 |
| 180 | 5 | 141251993 | 39971122; 29045549; 25782346; 21387833; 25065144 |
| 190 | 5 | 141252186 | 39971156; 29045585; 25782387; 21387873; 25065185 |
| 200 | 5 | 141252377 | 39971190; 29045620; 25782427; 21387914; 25065226 |
| 210 | 5 | 141252566 | 39971224; 29045654; 25782467; 21387954; 25065267 |
| 220 | 5 | 141252753 | 39971257; 29045689; 25782507; 21387994; 25065307 |
| 230 | 5 | 141252938 | 39971289; 29045723; 25782546; 21388033; 25065347 |
| 240 | 5 | 141253121 | 39971322; 29045756; 25782585; 21388072; 25065387 |
| 250 | 5 | 141253302 | 39971354; 29045789; 25782623; 21388110; 25065426 |
| 260 | 5 | 141253481 | 39971385; 29045822; 25782661; 21388149; 25065464 |
| 270 | 5 | 141253651 | 39971409; 29045854; 25782699; 21388186; 25065503 |
| 280 | 5 | 141253832 | 39971446; 29045886; 25782736; 21388224; 25065541 |
| 290 | 5 | 141253845 | 39971447; 29045888; 25782737; 21388228; 25065545 |

^a^ HEK293T cells were co-transfected with plasmids expressing MCP‑GFPdN and the PB2‑vMSL reporter for 20 hours, for live-cell imaging at 10-minute intervals from 60 to 300 m.p.i..

^b^ Total number containing vRNA puncta in cells and ^c^ their intensity of signals in Arbitrary Unit (A.U.) were determined by CAPS (1,2) .

**Supplementary Table S3. Primer sets for PCR amplification used to produce the templates for in vitro transcription of the viral RNA reference standards for the vRNA, cRNA, and mRNA of segments 5 (NP) and 6 (NA).**

| **Target** | | **Primer name** | **Sequence (5’ – 3’)** | **Position (nt)** |
| --- | --- | --- | --- | --- |
| NP | vRNA | seg5_1F | AGCAAAAGCAGGGTAGATAATCAC | 1-24 |
|  |  | T7_seg5_1565R | GGATCC TAATACGACTCACTATAGGG AGTAGAAACAAGGGTATTTTTCTT | 1565-1542 |
|  | cRNA | seg5_1565R | AGTAGAAACAAGGGTATTTTTCTT | 1565-1542 |
|  |  | T7_seg5_1F | GGATCC TAATACGACTCACTATAGGG AGCAAAAGCAGGGTAGATAATCAC | 1-24 |
|  | mRNA | seg5_dTR | TTTTTTTTTTT TTTTTCTTTAATTGTC | 1549-1534 |
|  |  | T7_seg5_1F | GGATCC TAATACGACTCACTATAGGG AGCAAAAGCAGGGTAGATAATCAC | 1-24 |
| NA | vRNA | seg6_1F | AGCGAAAGCAGGAGTTTAAATGAA | 1-24 |
|  |  | T7_seg6_1413R | GGATCC TAATACGACTCACTATAGGG AGTAGAAACAAGGAGTTTTTTGAA | 1413-1390 |
|  | cRNA | seg6_1413R | AGTAGAAACAAGGAGTTTTTTGAA | 1413-1390 |
|  |  | T7_seg6_1F | GGATCC TAATACGACTCACTATAGGG AGCGAAAGCAGGAGTTTAAATGAA | 1-24 |
|  | mRNA | seg6_dTR | TTTTTTTTTT TTTTTTGAACAAAC | 1398-1385 |
|  |  | T7_seg6_1F | GGATCC TAATACGACTCACTATAGGG AGCGAAAGCAGGAGTTTAAATGAA | 1-24 |

**Supplementary Table S4. Strand-specific primers for IAV detection for Flu-Stranded CRISPR-Cas12a assays and SYBR Green qPCR**

| Segment | RNA type | Step | Primer sequence (5’-3’)^#^ | CRISPR-CAS 12a | SYBR Green qPCR |
| --- | --- | --- | --- | --- | --- |
| NP | vRNA | RT^a^ | GGCCGTCATGGTGGCGAAT ACAGCCAAGTATACAGCCTAATC | ✓ | ✓ |
|  |  | PCR/ qPCR F | GGCCGTCATGGTGGCGAAT | ✓ | ✓ |
|  |  | PCR/ qPCR R | CCCAGTATCTGCTTCTCAGTTC | ✓ | ✓ |
| NP | cRNA | RT | GCTAGCTTCAGCTAGGCATC AGTAGAAACAAGGGTATTTTTCTTT | ✓ | ✓ |
|  |  | PCR/ qPCR F | GCTAGCTTCAGCTAGGCATC | ✓ | ✓ |
|  |  | PCR/ qPCR R | CGATCGTGCCCTCCTTTG | ✓ | ✓ |
| NP | mRNA | RT | CCAGATCGTTCGAGTCGT TTTTTTTTTTTTTTTTCTTTAATTGTC | ✓ | ✓ |
|  |  | PCR/ qPCR F | CCAGATCGTTCGAGTCGT | ✓ | ✓ |
|  |  | PCR/ qPCR R | CGATCGTGCCCTCCTTTG | ✓ | ✓ |
| NA | vRNA | RT | GGCCGTCATGGTGGCGAAT ACCATAATGACCGATGGCCCAAGT | ✓ | ✓ |
|  |  | PCR/ qPCR F | GGCCGTCATGGTGGCGAAT | ✓ | ✓ |
|  |  | PCR/ qPCR R | ACATCACTTTGCCGGTATCAGGGT | ✓ | ✓ |
| NA | cRNA | RT | GCTAGCTTCAGCTAGGCATC AGTAGAAACAAGGAGTTTTTTGAAC | ✓ | ✓ |
|  |  | PCR/ qPCR F | GCTAGCTTCAGCTAGGCATC | ✓ | ✓ |
|  |  | PCR/ qPCR R | TGAATAGTGATACTGTAGATTGGTCT | ✓ | ✓ |
| NA | mRNA | RT | CCAGATCGTTCGAGTCGT TTTTTTTTTTTTTTTTGAACAAACTAC | ✓ | ✓ |
|  |  | PCR/ qPCR F | CCAGATCGTTCGAGTCGT | ✓ | ✓ |
|  |  | PCR/ qPCR R | TGAATAGTGATACTGTAGATTGGTCT | ✓ | ✓ |

^a^ RT, Reverse Transcription

^#^, Tag sequences in primers are highlighted in gray

**Supplementary Table S5. Guide RNA (gRNA) sequences for strand-specific detection of IAV RNA species**

| **Target segment** | **RNA species** | **Guide RNA** | **Sequences** (5’-3’) |
| --- | --- | --- | --- |
| NP | vRNA | gRNA NP-v | TTTCCACTAGAGGAGTTCAAATTG |
|  | cRNA | gRNA NP-c/m | TTTAATTGTCGTACTCCTCTGCATT |
|  | mRNA | gRNA NP-c/m | TTTAATTGTCGTACTCCTCTGCATT |
| NA | vRNA | gRNA NA-v | TTTCAAGATCGAGAAGGGGAAGGTT |
|  | cRNA | gRNA NA-c/m | TTTGAACAAACTACTTGTCAATGGT |
|  | mRNA | gRNA NA-c/m | TTTGAACAAACTACTTGTCAATGGT |

**Supplementary Table S6. Strand-specific primers for TaqMan RT-qPCR detection of influenza A virus NP RNA**

| RNA type | Step | Primer sequence (5’-3’)^#^ |
| --- | --- | --- |
| vRNA | RT^a^ | GGCCGTCATGGTGGCGAAT GAATGGACGGAGAACAAGGATTGC |
|  | qPCR-F | GGCCGTCATGGTGGCGAAT |
|  | qPCR-R | CTCAATATGAGTGCAGACCGTGCT |
| cRNA | RT | GCTAGCTTCAGCTAGGCATC AGTAGAAACAAGGGTATTTTTCTTT |
|  | qPCR-F | GCTAGCTTCAGCTAGGCATC |
|  | qPCR-R | CGATCGTGCCCTCCTTTG |
| mRNA | RT | CCAGATCGTTCGAGTCGT TTTTTTTTTTTTTTTTCTTTAATTGTC |
|  | qPCR-F | CCAGATCGTTCGAGTCGT |
|  | qPCR-R | CGATCGTGCCCTCCTTTG |

^a^RT, Reverse Transcription

^#^, Tag sequences in primers are highlighted in gray

**Supplementary Table S7. Probe sequences used for strand-specific TaqMan RT-qPCR detection of influenza A virus RNA species**

| **Target segment** | **RNA type** | **Sequences (5’ FAM- 3’BHQ-1)** |
| --- | --- | --- |
| NP | vRNA | TGGATCAAGTGAGAGAGAGCCGGA |
|  | cRNA | TCGGAGACAATGCAGAGGAGTACGA |
|  | mRNA | TCGGAGACAATGCAGAGGAGTACGA |

**Supplementary Table S8. Primer sequences used to determine the expression levels of cellular genes by qPCR**

| Gene | Forward (5’-3’) | Reverse (5’-3’) |
| --- | --- | --- |
| RIG-I | CACCTCAGTTGCTGATGAAGGC | GTCAGAAGGAAGCACTTGCTACC |
| IFN-β | CTTGGATTCCTACAAAGAAGCAGC | TCCTCCTTCTGGAACTGCTGCA |
| ISG15 | ACAGCCATGGGCTGGGA | CCTTCAGCTCTGACACCGAC |
| IFIT3 | AGAAAAGGTGACCTAGACAAAGC | CCTTGTAGCAGCACCCAATCT |
| STAT1 | TGTATGCCATCCTCGAGAGC | AGACATCCTGCCACCTTGTG |
| OAS1 | AGGAAAGGTGCTTCCGAGGTAG | GGACTGAGGAAGACAACCAGGT |
| RNaseL | AAGGCTGTTCAAGAACTACACTTG | TGGATCTCCAGCCCACTTGATG |
| GAPDH | GAGTCAACGGATTTGGTCGT | GACAAGCTTCCCGTTCTCAG |

**Supplementary Table S9. Antiviral activity and cytotoxicity of interferons against IAV**

| Treatment | EC50^#^ (IU/mL) ± SD | CC50^&^ ( IU/mL) ± SD |
| --- | --- | --- |
| IFN-α | 5.57 ± 0.66 | > 1000 IU/ml |
| IFN-β | 3.13 ± 0.19 | > 1000 IU/ml |

^#^EC₅₀, half-maximal effective concentration;

^&^CC₅₀, half-maximal cytotoxic concentration

**References**

1.	Chiu, Y.-F., Sugden, A.U. and Sugden, B. (2013) Epstein-Barr viral productive amplification reprograms nuclear architecture, DNA replication, and histone deposition. *Cell host & microbe*, **14**, 607–618.

2.	Chiu, Y.-F., Huang, Y.-W., Chen, C.-Y., Chen, Y.-C., Gong, Y.-N., Kuo, R.-L., Huang, C.-G. and Shih, S.-R. (2022) Visualizing influenza a virus vRNA replication. *Frontiers in Microbiology*, **13**, 812711.
